# Paleolithic Diet—Effect on the Health Status and Performance of Athletes?

**DOI:** 10.3390/nu13031019

**Published:** 2021-03-21

**Authors:** Barbara Frączek, Aleksandra Pięta, Adrian Burda, Paulina Mazur-Kurach, Florentyna Tyrała

**Affiliations:** 1 Department of Sports Medicine and Human Nutrition, Institute of Biomedical Sciences, University School of Physical Education in Krakow, Jana Pawla II 78, 31-571 Cracow, Poland; aleksandrapieta@icloud.com (A.P.); paulina.mazurkurach@gmail.com (P.M.-K.); 2 Department of Econometrics and Operational Research, Cracow University of Economics, 31-510 Cracow, Poland; adrian.marek.burda07@gmail.com; 3 Department of Sports Dietetics, Gdansk University of Physical Education and Sport, 80-336 Gdansk, Poland; florentyna.tyrala@awf.gda.pl

**Keywords:** Paleolithic diet, health status, physical performance, randomized controlled trials, systematic review, meta-analysis

## Abstract

The aim of this meta-analysis was to review the impact of a Paleolithic diet (PD) on selected health indicators (body composition, lipid profile, blood pressure, and carbohydrate metabolism) in the short and long term of nutrition intervention in healthy and unhealthy adults. A systematic review of randomized controlled trials of 21 full-text original human studies was conducted. Both the PD and a variety of healthy diets (control diets (CDs)) caused reduction in anthropometric parameters, both in the short and long term. For many indicators, such as weight (body mass (BM)), body mass index (BMI), and waist circumference (WC), impact was stronger and especially found in the short term. All diets caused a decrease in total cholesterol (TC), low-density lipoprotein cholesterol (LDL-C), and triglycerides (TG), albeit the impact of PD was stronger. Among long-term studies, only PD cased a decline in TC and LDL-C. Impact on blood pressure was observed mainly in the short term. PD caused a decrease in fasting plasma (fP) glucose, fP insulin, and homeostasis model assessment of insulin resistance (HOMA-IR) and glycated hemoglobin (HbA1c) in the short run, contrary to CD. In the long term, only PD caused a decrease in fP glucose and fP insulin. Lower positive impact of PD on performance was observed in the group without exercise. Positive effects of the PD on health and the lack of experiments among professional athletes require longer-term interventions to determine the effect of the Paleo diet on athletic performance.

## 1. Introduction

The popularity of the Paleolithic diet has increased in recent years. Paleolithic nutrition is based on the principles of evolutionary biology with a focus on the low or moderate carbohydrate options available to the hunter–gatherers [1,2,3,4,5,6]. There is no expertise in the history or determination of what Paleolithic hominins ate. There are some papers supporting high intakes of animal food in the Paleolithic diet [1,5,7] and evidence that Paleolithic hominins ate plant foods [8,9,10,11,12]. It seems to be difficult to imagine one basic diet covering the entire period from 2.6 million to 10,000 years ago (when humans began to cultivate plants (predominantly cereal grains) and domesticate animals) and people living in a wide range of climates and geographic regions. What is more, there are still few differing viewpoints and controversies about what Paleolithic hominins really eat, the ability to replicate the Paleolithic diet in modern times, and the degree to which the human genetic profile has evolved to handle foods in the modern diet (based on an assumption that the modern human is not evolutionarily adapted to contemporary nutrition, which may result in a high incidence of diseases considered civilization-related) [2,5,13,14,15,16,17,18,19,20]. As detailed by many investigators, agricultural revolutions have introduced foods that were absent or negligible in the Paleolithic diet: refined cereal grains and their products, nonhuman mammalian milk and its products, energy-dense nutrient-poor foods (readily available and inexpensive refined carbohydrates, as well as separated fats and oils, all taking a wide variety of forms), and legumes [21,22,23,24,25,26,27,28]. The dietary approach, which is often referred to as the Paleo diet, targets the restriction of grains, dairy products, and all refined food items. The Paleo diet consists mainly of grass-fed and pasture-raised meats, vegetables, fruits, fungi, roots, and nuts, and excludes grains, legumes, and dairy products and limits refined sugars, starches, processed foods, and oils. In sum, the Paleo diet is relatively high in vitamins B (found in yeast, liver, and vegetables), D (found in mushrooms and liver), E (found in nuts, oils, vegetables, and fruits), and K (found in vegetables and liver), coenzyme Q10 (found in meat, fish, vegetables, and olive oil), alfa-lipoic acid (ALA) (found in organ meat, vegetables, and yeast), polyphenols (found in fruits, vegetables, herbs, nuts, tea, red wine, algae, coffee, chocolate, olives, and olive oil), carotenoids (from fruits, vegetables, olives, algae, and seafood), polyunsaturated fatty acids (from nuts, seeds, olive oil, seaweed, and fish), and elements such as selenium (found in nuts, fish and seafood) and zinc (found in nuts and seeds) [29,30,31,32]. As the diet is defined by the avoidance of particular food sources rather than a specific macronutrient distribution, there is a large degree of variation in the macronutrient composition of various Paleo diet interventions [33]. Kuipers proposes the following macronutrients distribution range: 25–29% (8–35%) energy from protein, 30–39% (20–72%) from fat, and 39–40% (19–48%) from carbohydrates. Cordain proposes, respectively, 19–35%, 28–58%, and 22–40% [34,35]. Authors of most Paleo interventions have embraced the potential of the ad libitum Paleo diet. Some researchers have found that a Paleo diet intervention yielded an improvement in glucose tolerance that appeared to be independent of energy intake and macronutrient distribution, prompting them to conclude that avoiding Western foods is more important than counting calories, fat, carbohydrate, or protein [28,36]. Currently, researchers evaluating the nutritional value of the Paleo diet classify it as a low-carbohydrate diet. On average, the authors estimate the following ratio of macronutrients: 35% energy from fats, 35% from carbohydrates, and 30% from protein (although no specific amount is considered to be the goal) [18,37]. According to this well-known division, it seems that the Paleo diet has a moderate amount of carbohydrates [38].

Recently, the Paleolithic diet became popular due to its possible health benefits. There are many scientific articles that evaluate the effect of the Paleo diet on health status—on diseases considered civilization-related, such as ischemic heart disease [28], blood lipid disorder [39], overweight or obesity [37,40,41,42,43,44,45,46,47,48,49], diabetes [28,42,43,44,50,51,52], and metabolic syndrome [53], and even on healthy, inactive adults [18,47,48,54,55,56]. Most, albeit not all, studies suggest that a PD has positive effects on body composition [28,37,39,40,41,42,43,44,45,46,47,48,49,52,53,54,55,56], insulin sensitivity and/or fasting blood glucose [18,28,37,41,42,44,46,48,52,53], blood lipids [18,37,39,41,42,44,46,47,48,49,53,54,55], and blood pressure [18,41,42,44,45,46,47,49,53,56]. Coupling dietary interventions with physical activity, which includes aerobic and resistance exercise, has been shown to benefit healthy adults or populations with type 2 diabetes mellitus (T2DM) [42,43,44,50]. Most researchers mentioned above used nutrition intervention ad libitum energy intake; there are only a few studies in which the authors have applied normoenergetic intake [18,52,53]. This resulted in, among other things, no reduction (or a non-significant effect), despite improvements in some metabolic indicators. That is why it is uncertain whether any of the positive health effects in these studies could also be on account of the accompanying weight loss as opposed to the composition of the Paleolithic-type diet per se [53].

The results of many clinical trials show evidence that low-carbohydrate diets (LCDs) with a high protein and fat content or even a ketogenic diet (KD) promote weight loss and improve the biomarkers of metabolic diseases, e.g., obese and type 2 diabetes [57]. The KD is successful for treating epilepsy in children [58,59]. On the other hand, in the long term, this diet may cause health problems. It increases the threat of kidney stones, blood lipid elevation, and bone fractures. As it is low in fiber, it also leads to constipation [60]. However, despite the positive effects of a low-carbohydrate/high-fat diet on body composition, no improvement in athlete performance has been observed [61,62]. A small number of KD (high fat, adequate protein, low carbohydrates) studies showed a minimal negative effect on power sports performance [61,63]. It has been suggested that benefits may be greatest for endurance sports requiring prolonged submaximal effort, including running and cycling, and perhaps also for field sports [58,64,65,66].

Many prominent CrossFit athletes advocate adherence to the Paleo diet, which has contributed to a recent boost in the popularity of the diet [55]. Only few studies have examined the effects of a PD with exercise on cardiorespiratory fitness in healthy or unhealthy adults [42,43,44,50,55]. To date, there is no scientific study on the effect of the Paleo diet on professional athletes. There are many studies determining the impact of a diet with very low amounts of carbohydrates (e.g., KD) on athletic performance, while there are no research trials with the use of a medium/moderate carbohydrate diet carried out in a group of athletes (which is now a very fashionable nutrition strategy willingly used by athletes). So far, no scientific studies have been carried out to check the use of the Paleo diet among athletes. It should be noted that the relatively small (but sufficient) amount of carbohydrates in the Paleo diet is increasingly used by athletes with the aim of using fat during exercise and saving carbohydrate reserves. That is why we became interested in this subject and decided to start research assessing the use of the Paleo diet under sports conditions. There are many positive features of the Paleo diet for athletes, where there is an emphasis on (1) protein and branched-chain amino acids (BCAAs), (2) eating an abundance of fruits and vegetables, (3) avoiding refined and processed carbohydrates to any high degree, and (4) health benefits [67]. Taking into consideration the objections to athletes’ use of the Paleo diet, we decided to review the current work on the impact of the Paleo diet on health status and physical performance.

The aim of this meta-analysis was to assess the effect of the Paleolithic diet (and healthy diets) on health status and physical capacity in professional athletes. The meta-analysis presents the results of the study overview regarding the impact of the Paleolithic diet (PD) on selected health indicators (body composition, lipid profile, blood pressure, and carbohydrate metabolism) and physical performance in the short and long term of nutritional intervention in healthy and unhealthy adults. The impact of the use of LCDs in sports was also researched.

## 2. Materials and Methods

### 2.1. Search Strategy

We searched EBSCO Discovery Service (http://web.b.ebscohost.com (accessed on 20 January 2020) databases, including MEDLINE, Sport Discuss with Full Text, Science Direct, and Scopus, from inception to July 2019. All studies found in the search were used. The research included randomized studies published in English; however, only 21 studies met the eligibility criteria. We used the MeSH terms related to the diet: “Paleolithic nutrition” OR “Paleolithic diet” OR “Paleolithic-type diet” OR “Paleo nutrition” OR “Paleo diet”.

### 2.2. Eligiblity Criteria

The retrieved studies were included in the review if they were an original article with a randomized control trial (RCT) design (parallel or crossover), irrespective of publication status, they involved humans. They assessed the effect of the Paleolithic diet on selected health indicators (body composition, lipid profile, blood pressure, or carbohydrate metabolism) and physical performance in adult athletes and in healthy (sedentary; physical inactive) or unhealthy adults, and their PD intervention consisted of lean meat, eggs, fish, seafood, nuts, fruits, and vegetables. Studies on diets containing dairy products, cereals, legumes, and added sugar or salt were excluded. Energy intake was ad libitum or normoenergetic. They had to assess (1) body mass and body composition, i.e., anthropometric parameters body mass (BM), body mass index (BMI), waist circumference (WC), and fat mass (FM) (kg and %); (2) lipid profile, i.e., total cholesterol (TC) (mg/dL), triglycerides (TG) (mg/dL), high-density lipoprotein cholesterol (HDL-C) (mg/dL), and low-density lipoprotein cholesterol (LDL-C) (mg/dL); (3) blood pressure (BP), i.e., systolic blood pressure (SBP) (mmHg), diastolic blood pressure (DBP) (mmHg), and heart rate (HR) (bpm); (4) carbohydrate metabolism, i.e., fasting plasma (fP) glucose (mmol/L), fP insulin (mmol/L), homeostasis model assessment of insulin resistance (HOMA-IR), and glycated hemoglobin (HbA1c) (%); (5) physical performance: VO_2_max (mL/kg/min), VO_2_max (L/min), and maximum workload (W max).

Studies were excluded if they (1) were non-RCTs, uncontrolled trials, observational studies (e.g., ecologic studies, cohort studies, case–control studies, case reports, case series, editorials, commentaries, letters to the editor, or qualitative research), conference papers, or publications available only in abstract form (no possible contact with the authors), (2) reported duplicate data from other included studies, (3) were conducted among children or adolescents aged younger than 18 y, or (4) did not report the targeted outcomes.

### 2.3. Data Extraction

**Figure 1:**
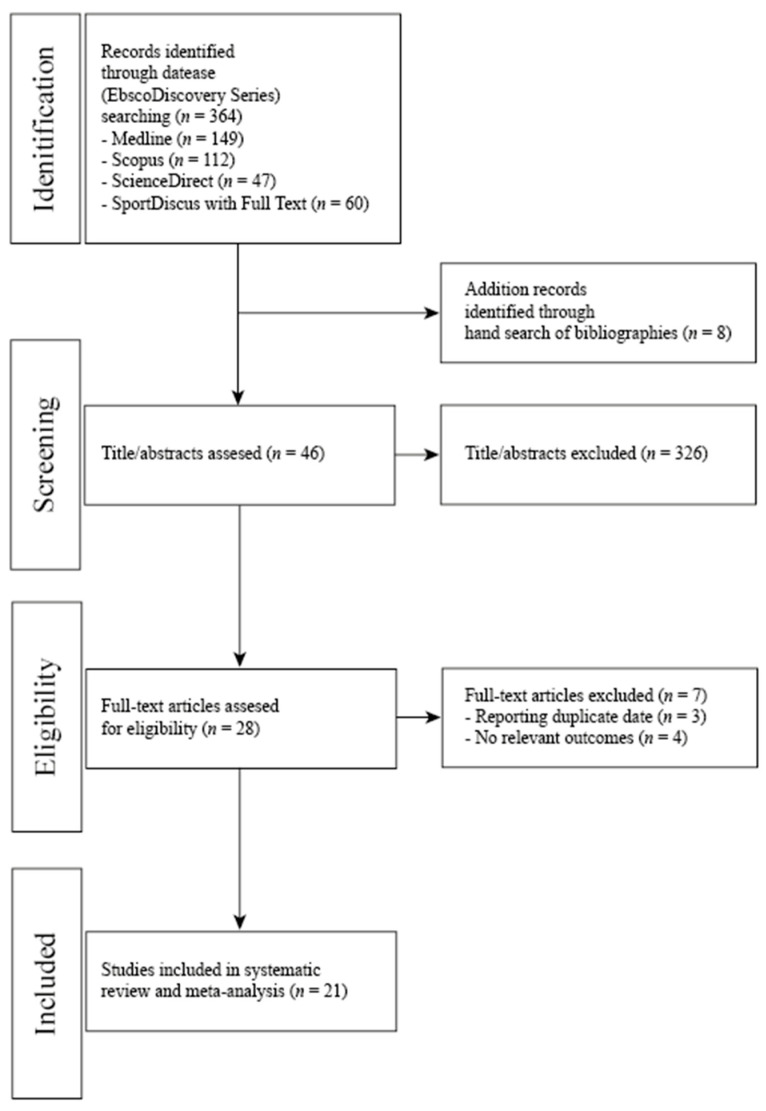
The search, screening, and selection process for the studies that were included in the review and meta-analyses.

We evaluated each article independently in the three main stages of the extraction process (Figure 1). Two independent researchers summarized the data, which were checked by two other authors. Firstly, article titles were screened, followed by abstracts and finally full texts for eligibility for inclusion in the systematic review and meta-analysis (first author’s name, year of publication, study design, study period, participants’ characteristics (n, age, sex, and health status), components of the dietary patterns consumed in the intervention and control groups, and the mean changes with corresponding SDs of measured outcomes in the intervention and control arms). Secondly full-text articles were assessed for eligibility. Disagreements were resolved by discussion between the investigators until a consensus was reached. All investigators agreed on the final exclusion if the study sought was unavailable or was only published in abstract form (Table 1).

We excluded some studies, such as that of Popp et al. [68], as overall results were not mentioned (only single results). Manousou et al.’s study [69] was also excluded as it reported the same values as that of Mellberg et al. [49]. The same characteristics of groups and similar outcomes were also noted in studies by Otten et al. [42], Otten et al. [43], and Stomby et al. [50]; thus, for all indicators reported in these studies, most outcomes from [43] (BMI and FM were included) were excluded, and all outcomes from [50] were excluded. While the majority of the included studies were classified as being of good quality, some studies had missing information or were not analyzed.

### 2.4. Statistical Analysis

For conducting meta-analysis, we applied package “Metafor” in an R environment (https://www.metafor-project.org/doku.php (accessed on 10 June 2020)). For all variables, we applied a random-effects model, because studies included in the meta-analysis were assumed to be a random selection from a larger population of studies, and the goal of analysis was to apply the results beyond the included studies. We assume the population of studies is a hypothetical population of an essentially infinite set of studies comprising all of the studies that have been conducted, that could have been conducted, or that may be conducted in the future. The “PDs” data group contains all studies concerning the Paleo diet, i.e., PD vs. a control diet (CD), PD pre (pre-intervention effect) vs. PD post (post-intervention effect), and PD vs. PD with an exercise program (PD-EX), and these are the results that we took into account when answering the question of whether the Paleo diet significantly influences the anthropometric or health indicators (because they are linked by the greatest number of interventions). Those studies cover the broadest sample of possible studies. Studies named as “PD” included only papers on PD vs. CD. Those results could be directly compared with control diet results. Here, we could directly compare impact of the Paleo diet with standard “healthy” diets. Furthermore, selected studies had various characteristic in terms of length (short-term studies between 2 weeks and 6 months and long-term studies over 6 months), investigated group characteristics (in terms of age, sex, BMI level, health status, nutrition strategy, or combined physical activity), and detailed characteristics of the Paleo diet (exact design, in particular in terms of calories). Finally, we assume that all studies share the same common effects. Thus, most criteria suggest that a random-effects model be used instead of a fixed-effects model [70]. Weights used in the estimation are equal to wᵢ = 1/(τ^2^ + vᵢ), with τ^2^ replaced by its estimate (again, this is the standard “inverse-variance” method for random-effects models). In the tables, we describe weighted average effects within group (control diet, Paleo diet, Paleo diet with exercise) with 95% confidence intervals, *p*-values of the effects, and the number of studies used for interference about the overall effect (Tables 2–10). Statistically significant results were defined as a *p*-value < 0.1. A detailed description can be found below each table. Forest plot graphs were analyzed to examine the overall effect and publication bias. The mean effect described in the publication and its 95% confidence bands are presented. Finally, the results for indicators from fewer than five studies [70] should be interpreted very cautiously, especially because there is no common effect in the population and studies cover a very similar sample and research period.

Due to the large number of analyses, in Appendix A, we present forest plots of the effect of a PDs (both PD vs. CD and PD pre vs. post; PD vs. PD + EX), PD (PD vs. CD studies) and CD on indicators of health status (Figure A1, Figure A2, Figure A3, Figure A4, Figure A5, Figure A6, Figure A7, Figure A8, Figure A9, Figure A10, Figure A11, Figure A12, Figure A13, Figure A14, Figure A15, Figure A16, Figure A17, Figure A18, Figure A19, Figure A20, Figure A21, Figure A22, Figure A23, Figure A24, Figure A25, Figure A26, Figure A27, Figure A28, Figure A29, Figure A30, Figure A31, Figure A32, Figure A33, Figure A34, Figure A35, Figure A36, Figure A37, Figure A38, Figure A39, Figure A40, Figure A41, Figure A42, Figure A43, Figure A44, Figure A45, Figure A46, Figure A47, Figure A48, Figure A49, Figure A50, Figure A51, Figure A52, Figure A53 and Figure A54). All results presented in the forest plots are in the main text of this paper in tables and in the description of the results. We did not present forest plots that included fewer than three studies (in all: PDs, PD, and CD).

## 3. Results

### 3.1. Characteristics of Included Studies and Search Results

The characteristics of the study participants, research details, and evaluated outcomes are shown in Table 1. In total, 700 subjects were included in the meta-analysis. All studies were conducted in adult populations. The average age of study participants ranged from 30.0 ± 10.0 years [56] to 66.0 ± 6.0 years [51] in the Paleolithic diet group, and similar values were observed in the control group. Studies included women, men, or both. In a few studies, the information about the sex of study participants was not indicated [28,42,51]. Four studies included subjects that had type 2 diabetes mellitus and or were obese [51,52], eleven studies included overweight or obese postmenopausal women [37,40,41,42,43,44,45,46,47,48,49], one study included subject with hypercholesterolemia [39], one study recruited subjects with ischemic heart disease plus either glucose intolerance or type 2 diabetes [28], one study was conducted in subjects with at least two characteristics of the metabolic syndrome [53], and four studies recruited healthy but inactive adults [18,55,56,71]. We examined the significance of the change in PD and healthy diets (CDs) based on the Nordic Nutrition Recommendation (NNR) [40,45,46,49], the Dutch Health Council [53], the Australian Guide to Healthy Eating (AGHE) [54], the American Diabetes Association (ADA) [52], the American Heart Association (AHA) [39], the Mediterranean diet [28], a conventional low-fat diet (LFD) [41], and the so-called “diabetes diet” [51]. There were 14 studies including the analysis of PD vs. CD [28,39,40,41,42,43,44,45,46,47,48,49,50,51,52,53,54,56], 3 studies on PD pre vs. post [18,37,71], and 3 on PD vs. PD + EX (physical activity, i.e., combined aerobic and resistant training) [42,44,55]. Interventions ranged from 2 weeks to 24 months. Sixteen studies assessing the impact of the Paleo diet on body composition, i.e., “anthropometric parameters”—body mass (BM), body mass index (BMI), waist circumference (WC), and fat mass (FM) (kg and %), were analyzed. The changes in the lipid profile determined by total cholesterol (TC) (mg/dL), triglycerides (TG) (mg/dL), high-density lipoprotein cholesterol (HDL-C) (mg/dL), and low-density lipoprotein cholesterol (LDL-C) (mg/dL) were analyzed based on the analysis of 14 studies. Analyzing the results of 12 studies, the influence of the Paleo diet on blood pressure, i.e., systolic blood pressure (SBP) (mmHg), diastolic blood pressure (DBP) (mmHg), and HR (bpm), were assessed. Twelve studies assessing the influence of PD on carbohydrate metabolism, i.e., fasting plasma (fP) glucose (mmol/L), fP insulin (mmol/L), homeostasis model assessment of insulin resistance (HOMA-IR), and glycated hemoglobin (HbA1c) (%), were analyzed. Finally, physical performance, i.e., VO_2_max (mL/kg/min), VO_2_max (L/min), and maximum workload (W max), were assessed in three studies.

### 3.2. Effect of the Paleolithic Diet on Body Composition—Anthropometric Parameters (Body Mass, Body Mass Index, Waist Circumference, Fat Mass, and Fat Free Mass) in the Short (up to 6 Months) and Long (over 6 Months) Term

**Figure A1:**
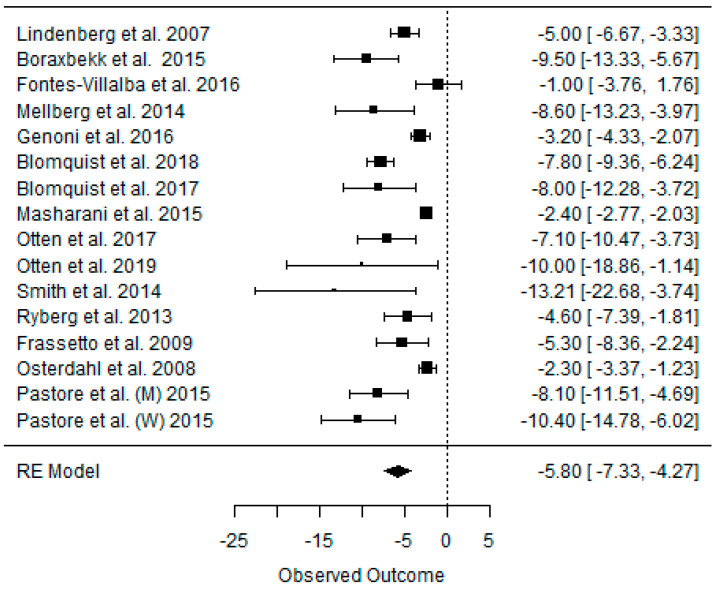
Forest plot of the effect of a PDs on BM (kg) in the short term (weighted mean difference).

**Figure A2:**
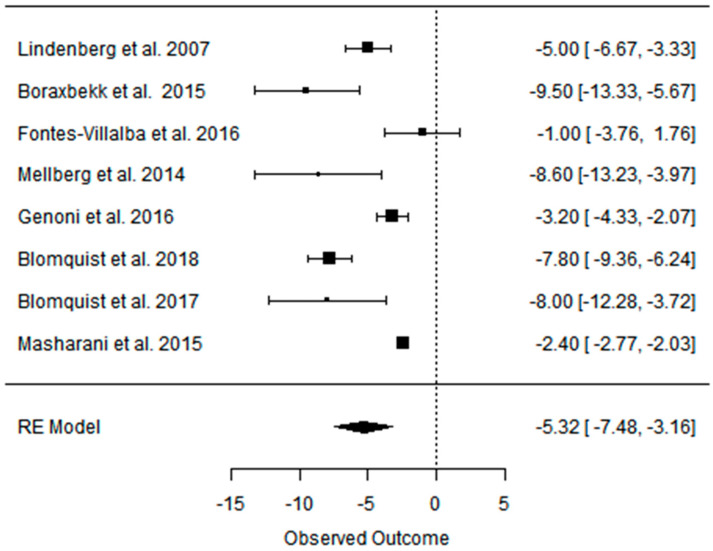
Forest plot of the effect of PD on BM (kg) in the short term (weighted mean difference).

**Figure A3:**
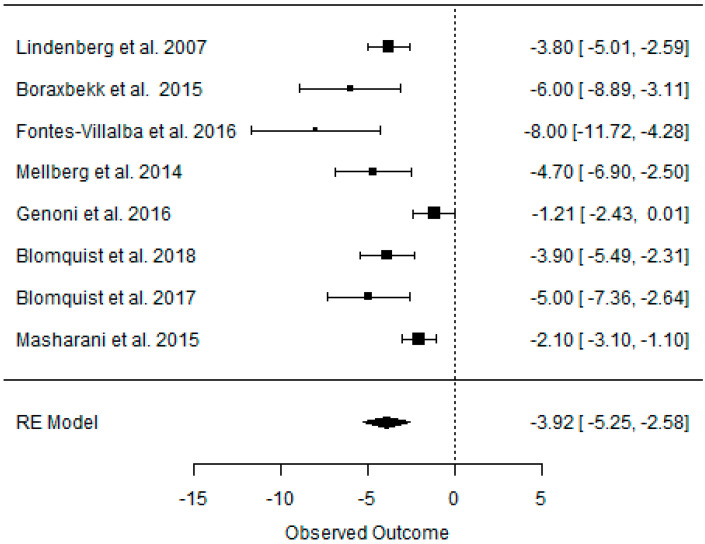
Forest plot of the effect of a CD on BM (kg) in the short term (weighted mean difference).

**Figure A4:**
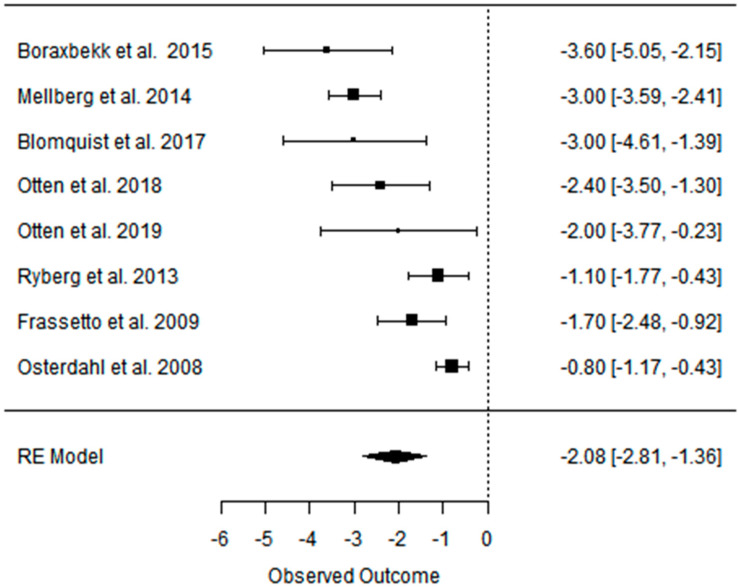
Forest plot of the effect of a PDs on BMI in the short term (weighted mean difference).

**Figure A5:**
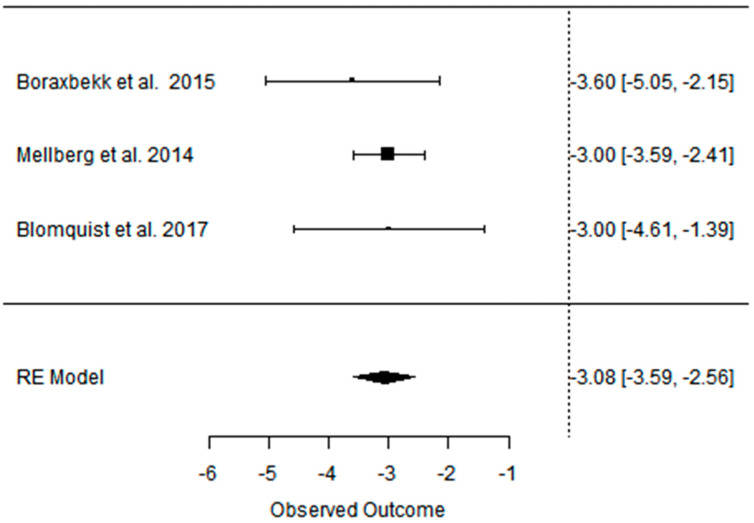
Forest plot of the effect of PD on BMI in the short term (weighted mean difference).

**Figure A6:**
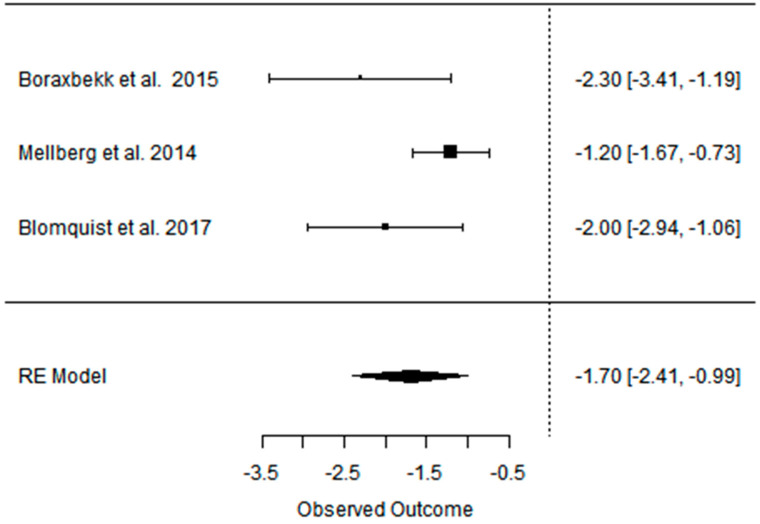
Forest plot of the effect of a CD on BMI in the short term (weighted mean difference).

**Figure A7:**
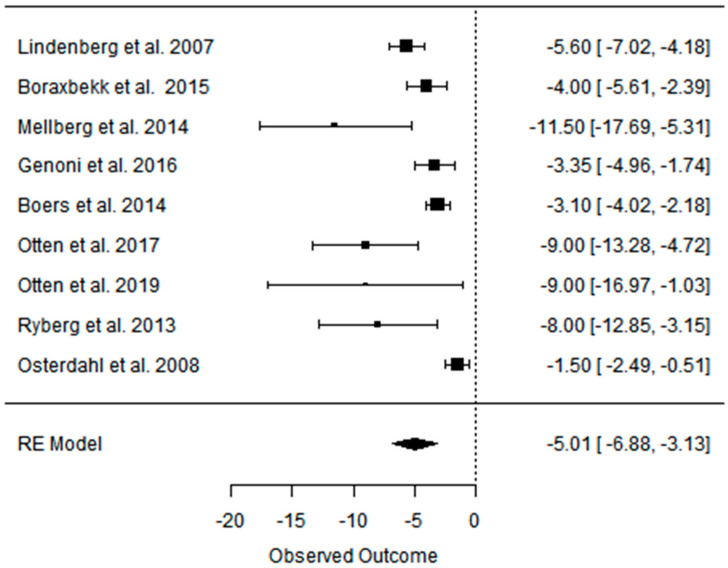
Forest plot of the effect of a PDs on waist circumference (cm) in the short term (weighted mean difference).

**Figure A8:**
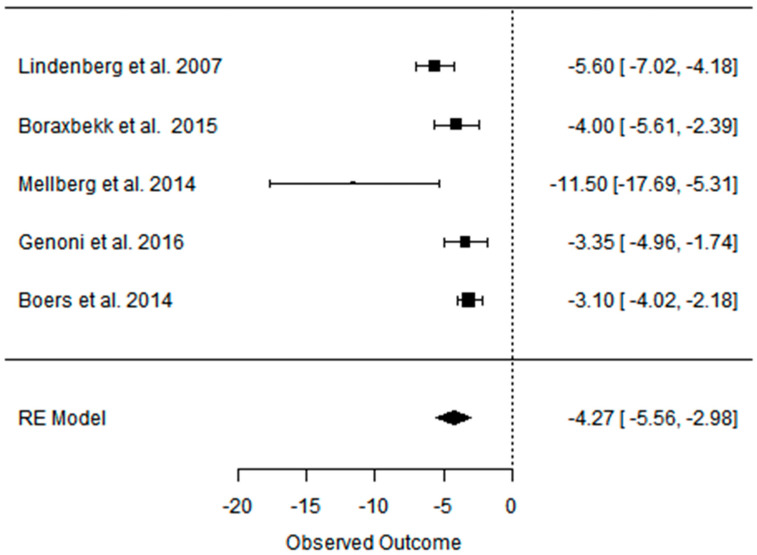
Forest plot of the effect of PD on waist circumference (cm) in the short term (weighted mean difference).

**Figure A9:**
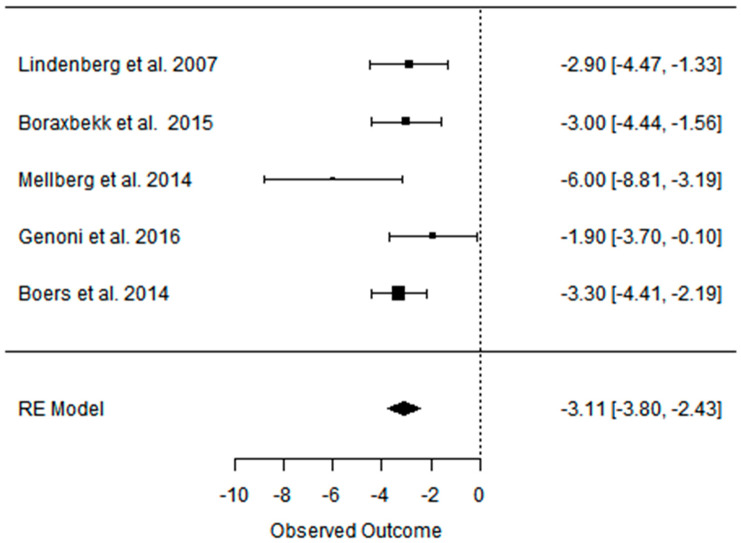
Forest plot of the effect of a CD on waist c (cm) in the short term (weighted mean difference).

The impact of the Paleo diet on body mass in all independently investigated studies (16) [18,28,37,39,40,42,44,47,48,49,51,52,54,55,56] (Figure A1) with this indicator was on average −5.8 kg (95% confidence bands: −4.3; −7.3) and was statistically significant at the 0.001 confidence level. We observed similar results, with a slightly lower effect (−5.3 kg, 95% confidence bands: −3.2; −7.5) in studies on PD vs. CD (eight studies) [28,40,47,48,49,51,52,54] (Figure A2). Simultaneously, CDs in these studies (eight) also significantly reduced the weight of the participants, albeit the average effect was substantially smaller (−3.9 kg with 95% confidence bands: −2.6; −5.3) [28,40,47,48,49,51,52,54] (Figure A3). The impact of PD on BMI in all independently investigated studies (eight) with this indicator was on average −2.1 (95% confidence bands: −1.4; −2.8) and was statistically significant at the 0.001 confidence level [18,37,40,43,44,47,49,56] (Figure A4). We observed similar results, with a higher effect (−3.1; 95% confidence bands: −2.6; −3.6), in studies on PD vs. CD (three studies) [40,47,49] (Figure A5). Simultaneously, the CDs in these studies (three) also significantly reduced the BMI of the participants, albeit the average effect was substantially smaller (−1.7 with 95% confidence bands: −1.0; −2.4) [40,47,49] (Figure A6). The impact of PD on waist circumference in all independently investigated studies (nine) with this indicator was on average −5.0 cm (95% confidence bands: −3.1; −6.9) and was statistically significant at the 0.001 confidence level [28,37,40,42,44,49,53,54,56] (Figure A7). We observed similar results, with a slightly lower effect (−4.2cm, 95% confidence bands: −3.0; −5.6), in studies on PD vs. CD (five studies) [28,40,49,53,54] (Figure A8). Simultaneously, the CDs in these studies (five) also significantly reduced the waist circumference of the participants, albeit the average effect was substantially smaller (−3.1 cm with 95% confidence bands: −2.4; −3.8) [28,40,49,53,54] (Figure A9). The impact of the Paleo diet on fat mass (kg) in all independently investigated studies (three) with this indicator was on average −4.5 kg (95% confidence bands: −1.6; −7.5) and was statistically significant at the 0.003 confidence level [43,49,54]. We observed a very similar effect (−4.1 kg, 95% confidence bands: 0.4; −8.6) in studies on PD vs. CD (two studies), though it was statistically significant only at the 0.1 significance level [49,54]. While the CDs in these studies (two) also substantially reduced fat mass (−2.1 kg), this effect was not statistically significant [49,54]. The impact of the Paleo diet on fat mass as a % of total mass in all independently investigated studies (four) with this indicator was on average −2.4% (95% confidence bands: −1.2; −3.6) and was statistically significant at the 0.001 confidence level [42,46,54,55]. We observed a slightly higher impact of the Paleo diet on FM as a % of total mass, which was statistically significant at the 0.1 confidence level (−2.6%, 95% confidence bands: 0.1; −5.2), in studies on PD vs. CD (two studies) [46,54]. While participants of those studies who used CDs also reduced their fat in terms of a % of total mass (on average by 0.3%), this decline was not statistically significant (95% confidence bands: 1.3; −1,9) [46,54] (Table 2).

**Figure A10:**
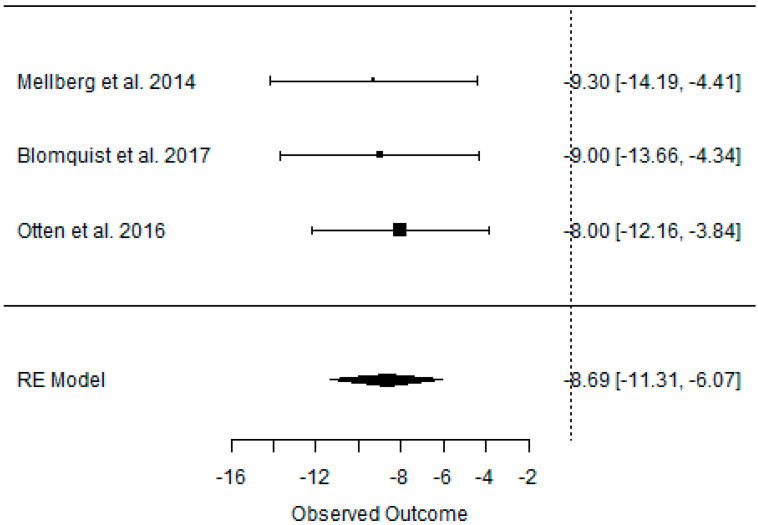
Forest plot of the effect of a PD on BM (kg) in the long term (weighted mean difference).

**Figure A11:**
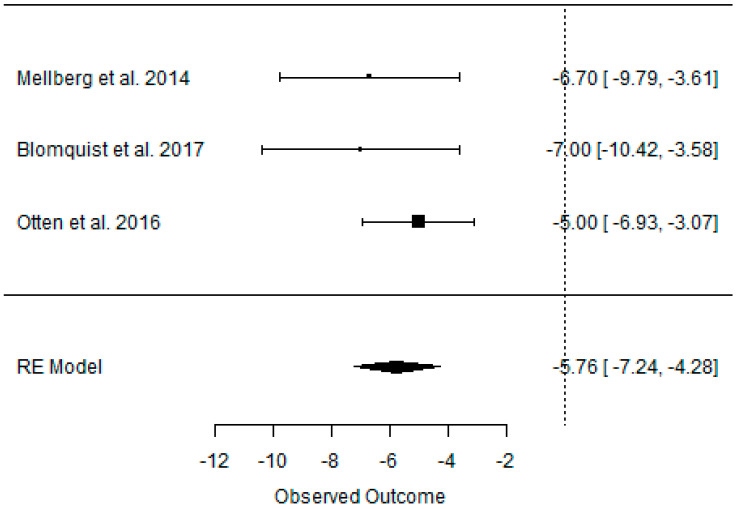
Forest plot of the effect of a CD on BM (kg) in the long term (weighted mean difference).

**Figure A12:**
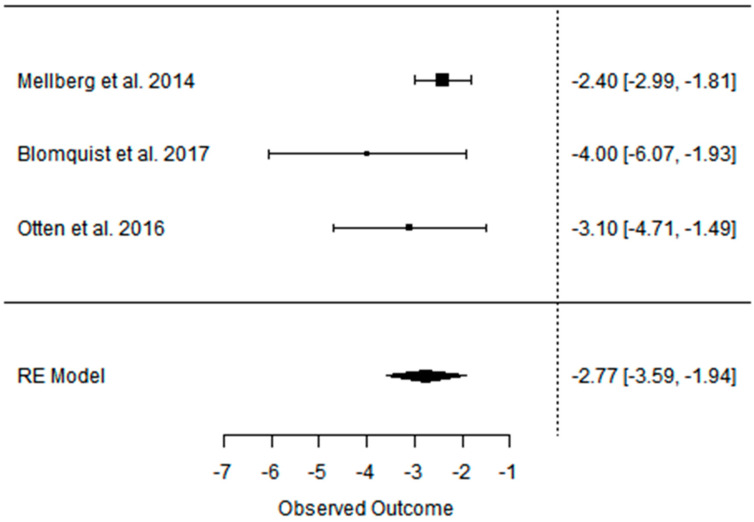
Forest plot of the effect of a PD on BMI in the long term (weighted mean difference).

**Figure A13:**
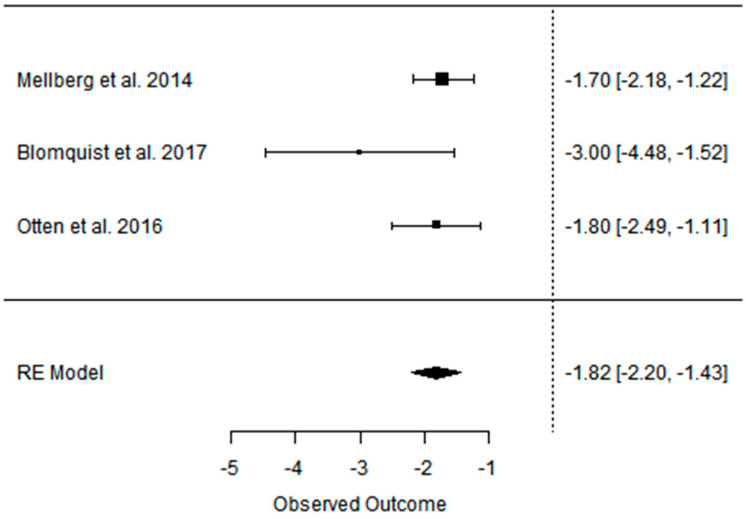
Forest plot of the effect of a CD on BMI in the long term (weighted mean difference).

All studies where the long-term impact of the Paleo diet on body composition indicators was investigated are PD vs. CD studies. Thus, in what follows, we describe only the impact of PD and CD, without differentiating the type of study. The long-term impact of the Paleo diet on weight was on average −8.7 kg (95% confidence bands: −6.1; −11.3) and was statistically significant at the 0.001 confidence level [41,47,49] (Figure A10). Simultaneously, the CDs also significantly reduced the weight of the participants in the long term, albeit the average effect was substantially smaller (−5.8 kg with 95% confidence bands: −4.3; −7.2) [41,47,49] (Figure A11). The long-term impact of the Paleo diet (three studies) on BMI was on average −2.8 (95% confidence bands: −1.9; −3.6) and was statistically significant at the 0.001 confidence level [41,47,49] (Figure A12). Simultaneously, the control diets (three studies) also significantly reduced the weight of the participants in the long term, albeit the average effect was substantially smaller (-1.8 with 95% confidence bands: −1.4; −2.2) [41,47,49] (Figure A13). The long-term impact of the Paleo diet (two) on waist circumference was on average −12.1 cm (95% confidence bands: −7.6; −16.6) and was statistically significant at the 0.001 confidence level [41,49]. Simultaneously, the control diets (two) also significantly reduced the waist of the participants in the long term, albeit the average effect was substantially smaller (−10.9 cm with 95% confidence bands: −7.6; −14.1) [41,49]. The long-term impact of PD (two) on fat mass was on average −5.5 kg (95% confidence bands: −3.4; −7.5) and was statistically significant at the 0.001 confidence level [41,49]. Simultaneously, the CDs (two) also significantly reduced the fat mass of the participants in the long term, albeit the average effect was substantially smaller (−4.5 kg with 95% confidence bands: −2.9; −6.0) [41,49]. The long-term impact of the Paleo diet (two) on fat (as a % of total mass) was on average −2.7% (95% confidence bands: −1.3; −4.1) and was statistically significant at the 0.001 confidence level [41,46]. Simultaneously, the control diets (two) also significantly reduced the fat mass of the participants, with a similar average effect (−2.6 with 95% confidence bands: −0.8; −4.5) [41,46] (Table 3).

### 3.3. Effect of the Paleolithic Diet on Lipid Profile (Total Cholesterol, Triglycerydes, HDL-C, and LDL-C in the Short (up to 6 Months) and Long (over 6 Months) Term

**Figure A14:**
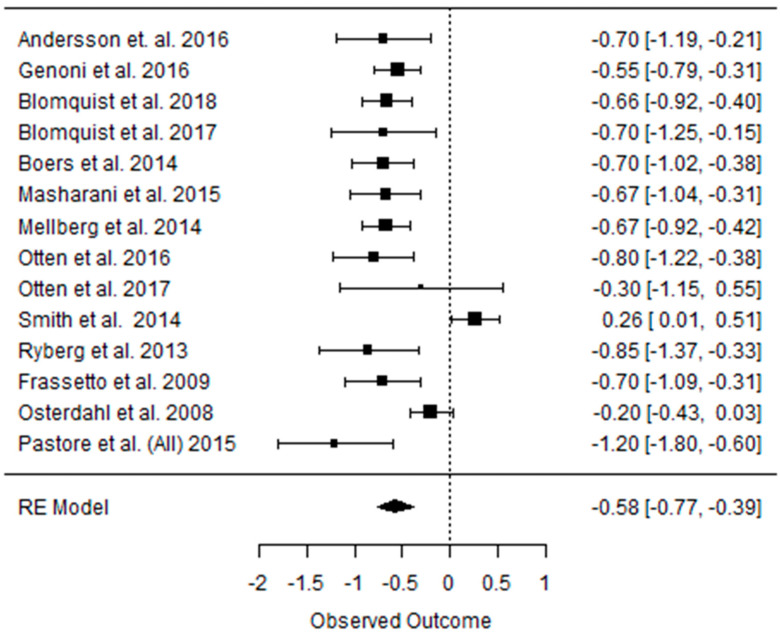
Forest plot of the effect of a PDs on TC in the short term (weighted mean difference).

**Figure A15:**
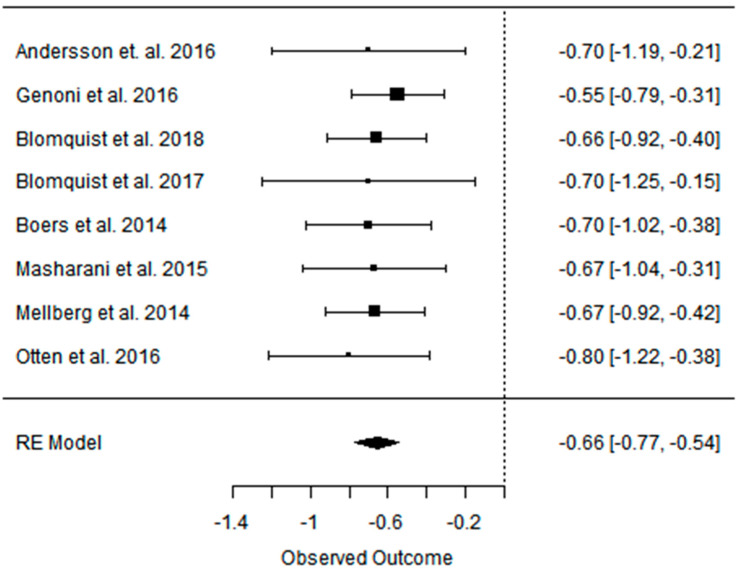
Forest plot of the effect of PD on TC in the short term (weighted mean difference).

**Figure A16:**
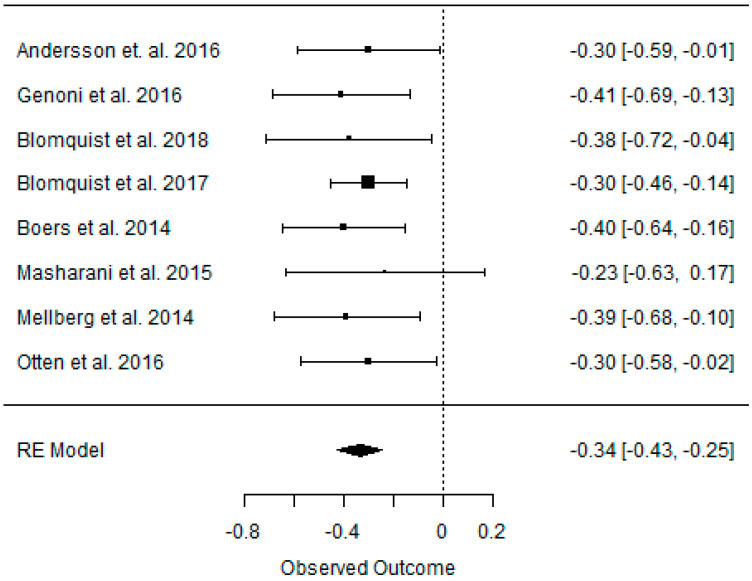
Forest plot of the effect of a CD on TC in the short term (weighted mean difference).

**Figure A17:**
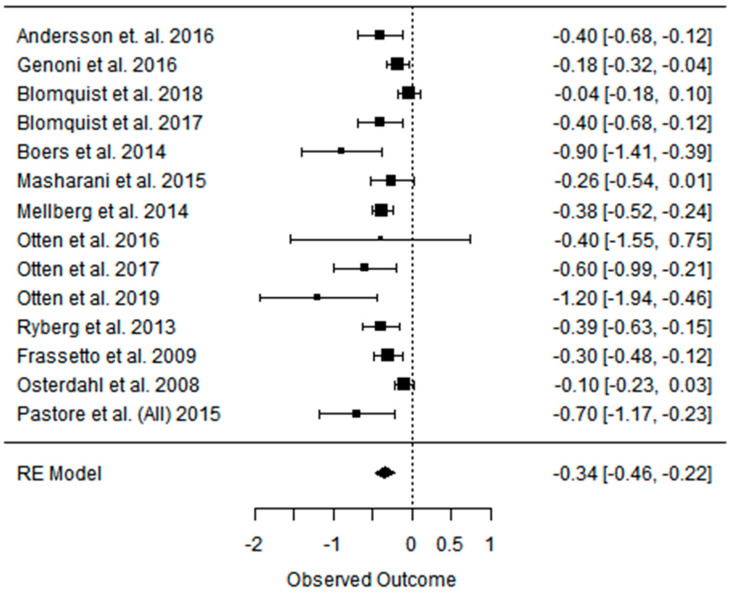
Forest plot of the effect of a PDs on TG in the short term (weighted mean difference).

**Figure A18:**
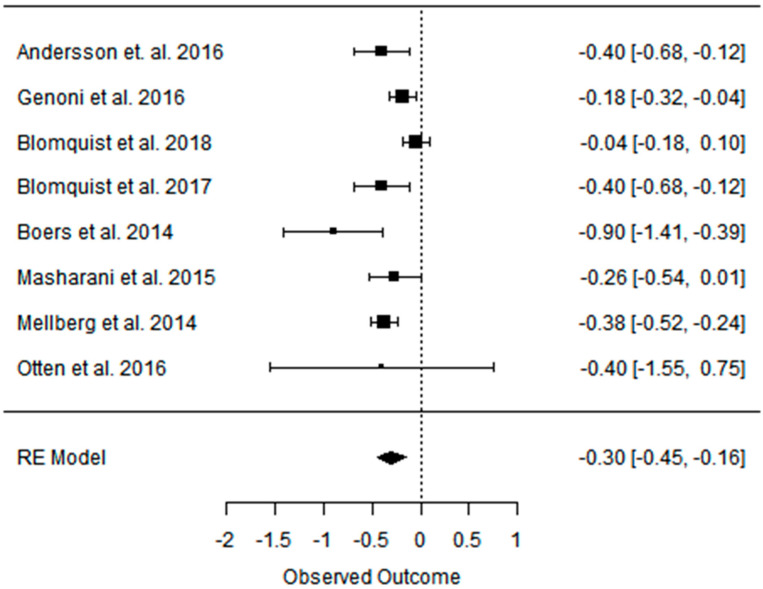
Forest plot of the effect of PD on TG in the short term (weighted mean difference).

**Figure A19:**
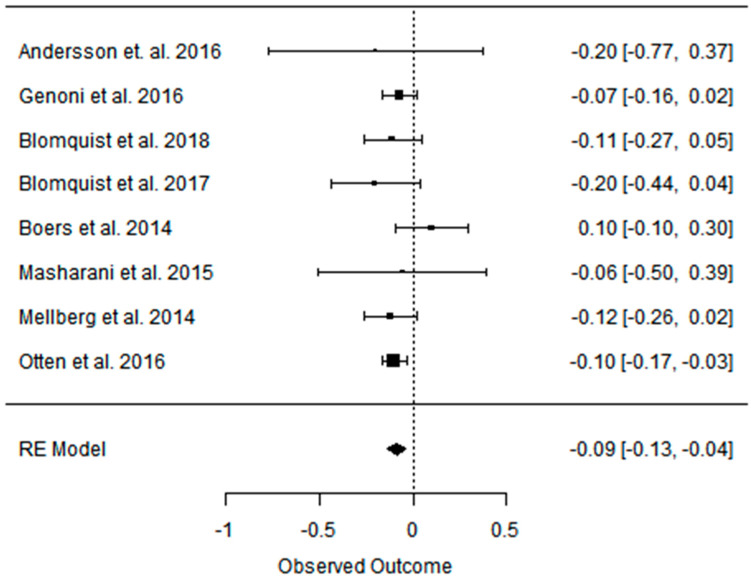
Forest plot of the effect of a CD on TG in the short term (weighted mean difference).

**Figure A20:**
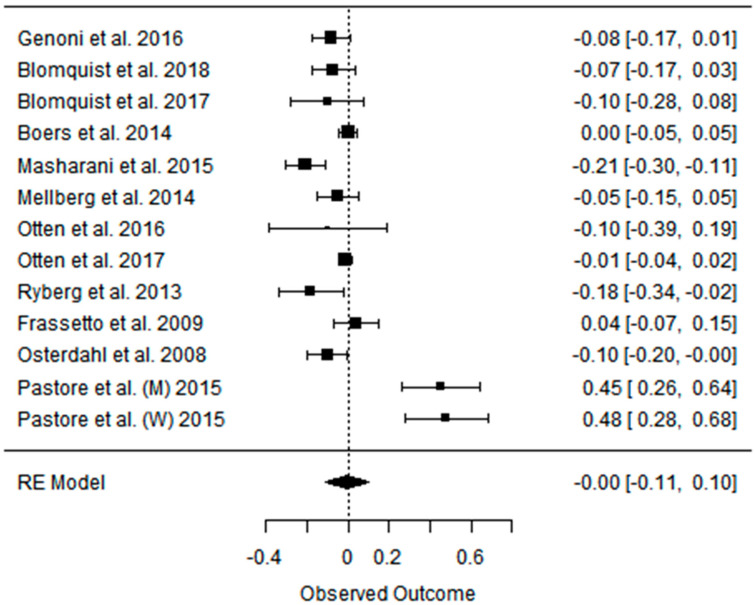
Forest plot of the effect of a PDs on HDL-C in the short term (weighted mean difference).

**Figure A21:**
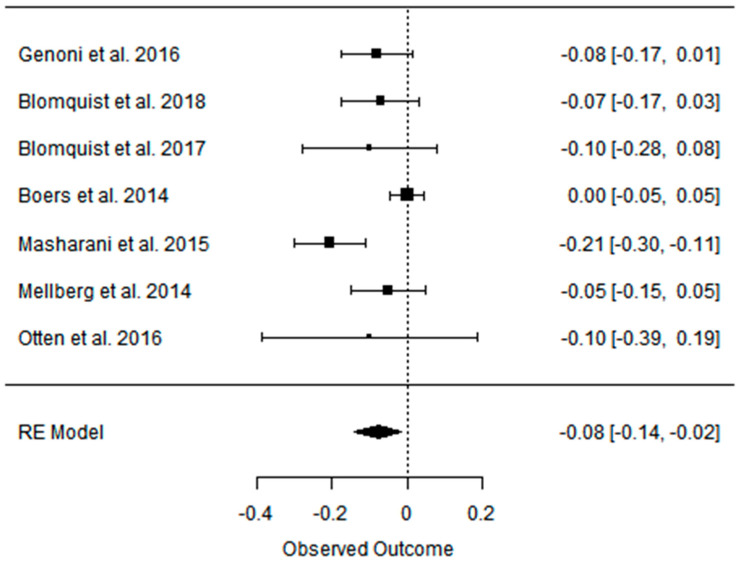
Forest plot of the effect of PD on HDL-C in the short term (weighted mean difference).

**Figure A22:**
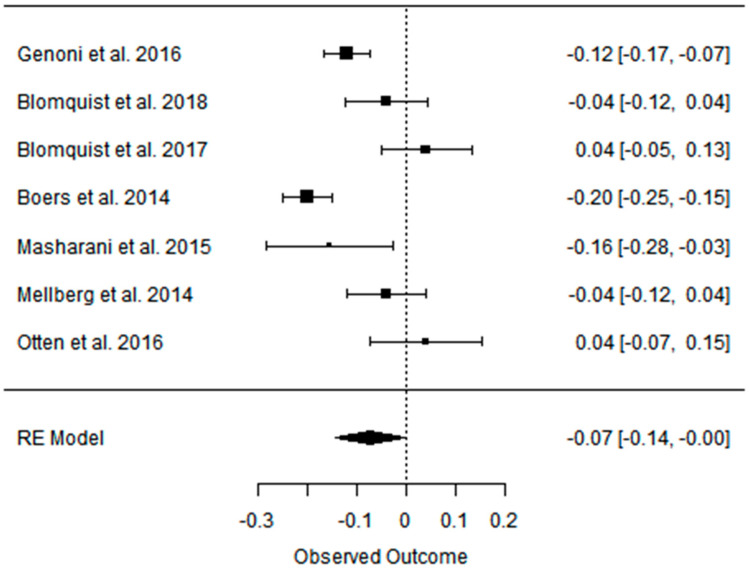
Forest plot of the effect of a CD on HDL-C in the short term (weighted mean difference).

**Figure A23:**
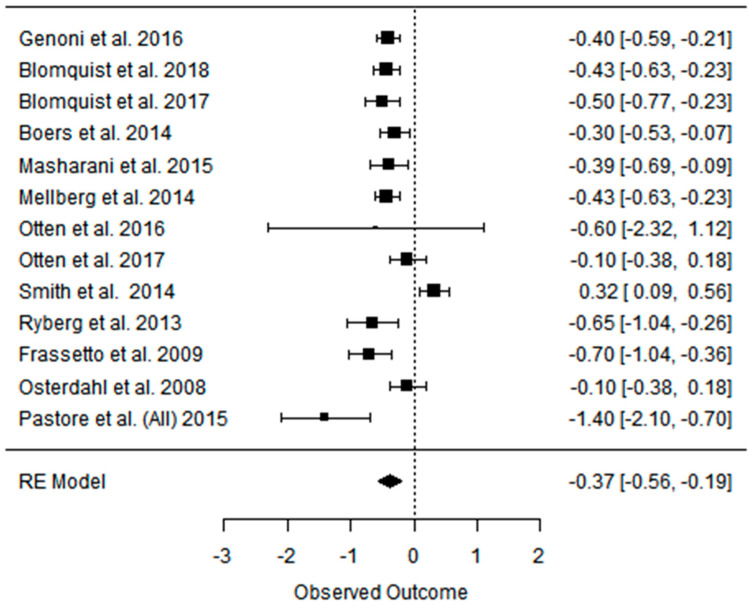
Forest plot of the effect of a PDs on LDL-C in the short term (weighted mean difference).

**Figure A24:**
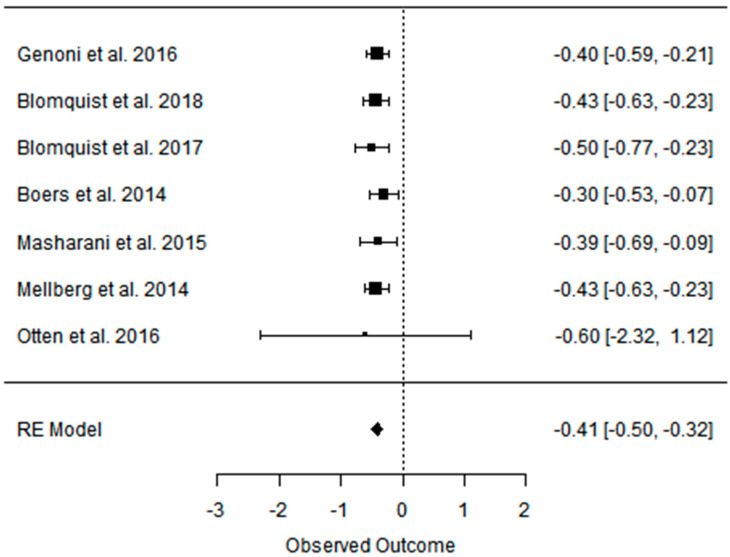
Forest plot of the effect of PD on LDL-C in the short term (weighted mean difference).

**Figure A25:**
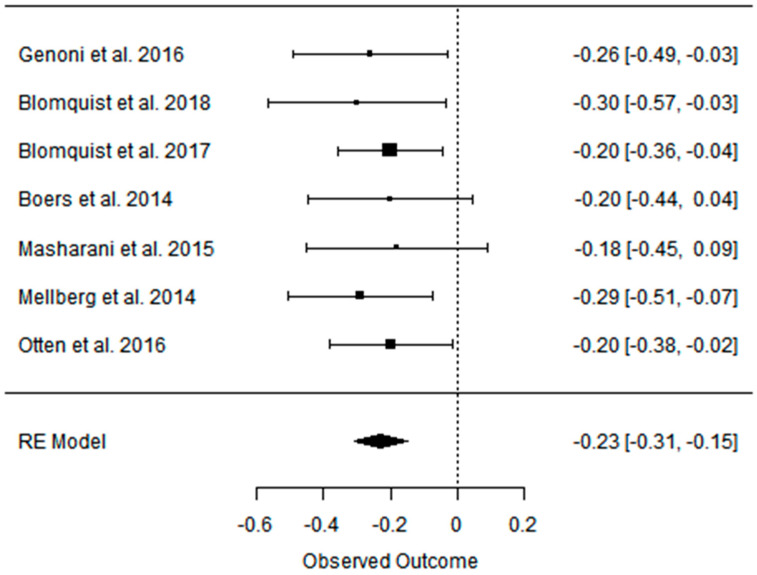
Forest plot of the effect of a CD on LDL-C in the short term (weighted mean difference).

The impact of the Paleo diet on TC in all independently investigated studies (14) with this indicator was on average −0.6 mg/dL (95% confidence bands: −0.4; −0.8) and was statistically significant at the 0.001 level [18,37,39,41,42,44,45,47,48,49,52,53,54,55,56] (Figure A14). We observed a similar effect, which was statistically significant also at the 0.001 significance level (on average −0.7 mg/dL, 95% confidence bands: −0.5; −0.8), in studies Paleo diet vs. control diet (eight studies) [41,45,47,48,49,52,53,54] (Figure A15). However, the control diets in these studies (eight) also had a statistically significant impact on TC [41,45,47,48,49,52,53,54] (Figure A16). The impact of the Paleo diet on TG in all independently investigated studies (14) with this indicator was on average −0.35 mg/dL (95% confidence bands: −0.2; −0.5) and was statistically significant at the 0.001 level [18,37,39,41,42,44,45,47,48,49,52,53,54,56] (Figure A17). We observed a similar effect, which was statistically significant at the 0.001 level (on average −0.30 mg/dL, 95% confidence bands: 0.2; −0.4), in studies on PD vs. CD (eight studies) [41,45,47,48,49,52,53,54] (Figure A18). However, the control diets in these studies (eight) had a statistically significant impact on TG as well [41,45,47,48,49,52,53,54] (Figure A19). The impact of the Paleo diet on HDL-C in all independently investigated studies (13) with this indicator was on average near zero and not statistically significant [18,37,39,41,42,44,45,47,48,49,52,53,54,56] (Figure A20). However, the impact of the Paleo diet on HDL-C in studies on PD vs. CD (seven studies) was statistically significant at the 0.05 level (−0.08 mg/dL, 95% confidence bands: −0.02; −0.14) [41,47,48,49,52,53,54] (Figure A21). Similarly, the control diets in these studies (seven) also had a statistically significant impact on HDL-C at the 0.05 significance level (−0.07 mg/dL, 95% confidence bands: 0; −0.14) [41,45,47,48,49,52,53,54] (Figure A22). The impact of the Paleo diet on LDL-C in all independently investigated studies (13) with this indicator was on average −0.37 mg/dL. (95% confidence bands: −0.19; −0.56) and was statistically significant at the 0.001 level [18,37,39,40,41,42,43,44,45,46,47,48,49,50,51,52,53,54,56] (Figure A23). We observed a slightly higher effect, which was statistically significant also at the 0.001 significance level (on average −0.41 mg/dL, 95% confidence bands: −0.32; −0.50), in studies on PD vs. CD (seven studies) [41,47,48,49,52,53,54] (Figure A24). The CDs in these studies (seven) also had a statistically significant impact on LDL-C (on average −0.23 mg/dL, 95% confidence bands: 0.15; −0.31) [41,47,48,49,52,53,54] (Figure A25) (Table 4).

**Figure A26:**
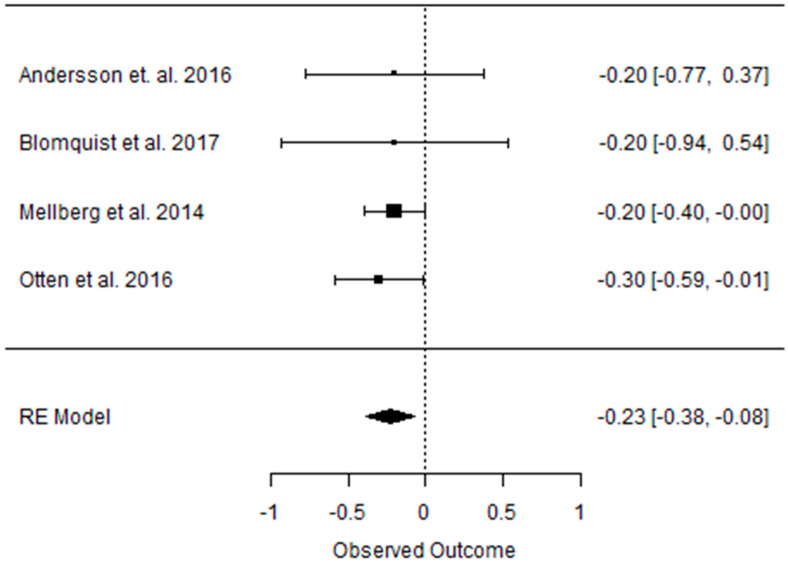
Forest plot of the effect of a PD on TC in the long term (weighted mean difference).

**Figure A27:**
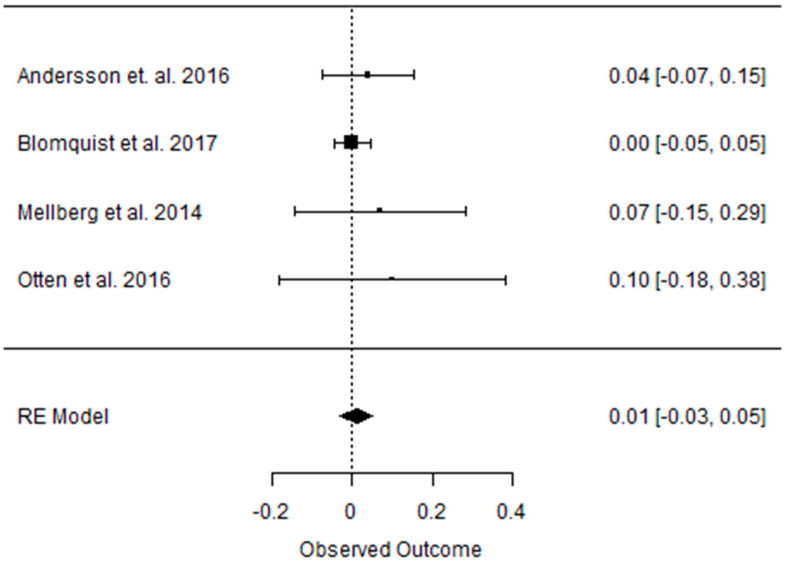
Forest plot of the effect of a CD on TC in the long term (weighted mean difference).

**Figure A28:**
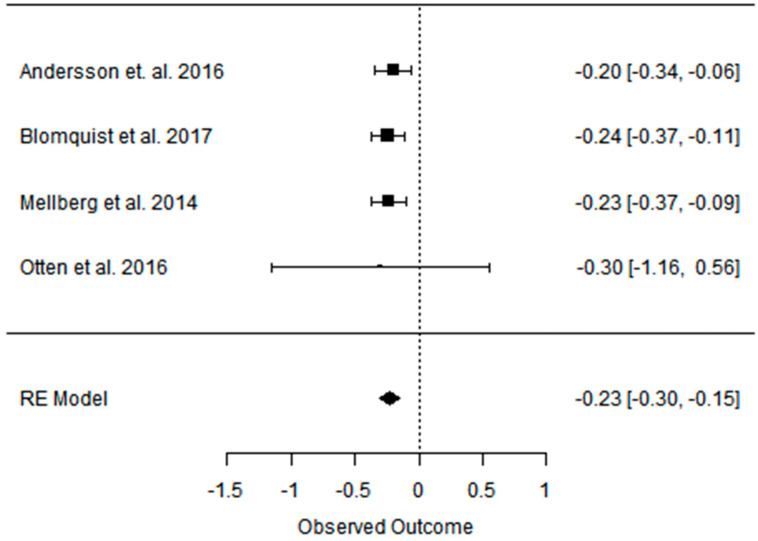
Forest plot of the effect of a PD on TG in the long term (weighted mean difference).

**Figure A29:**
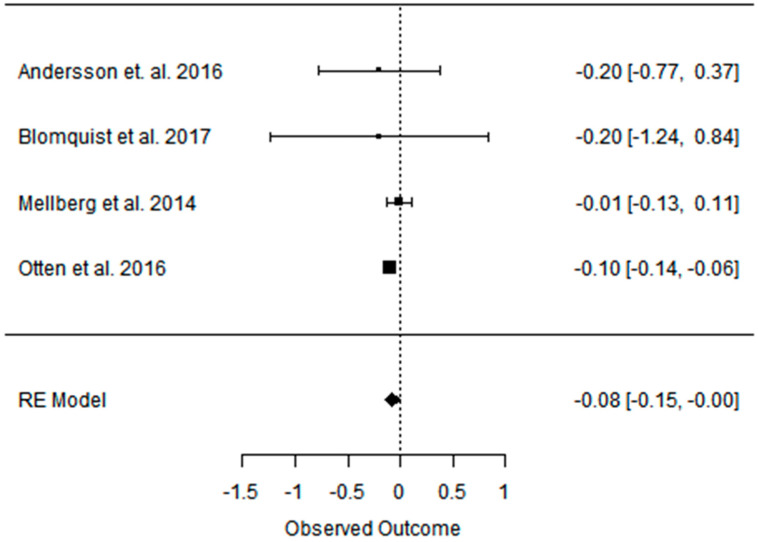
Forest plot of the effect of a CD on TG in the long term (weighted mean difference).

**Figure A30:**
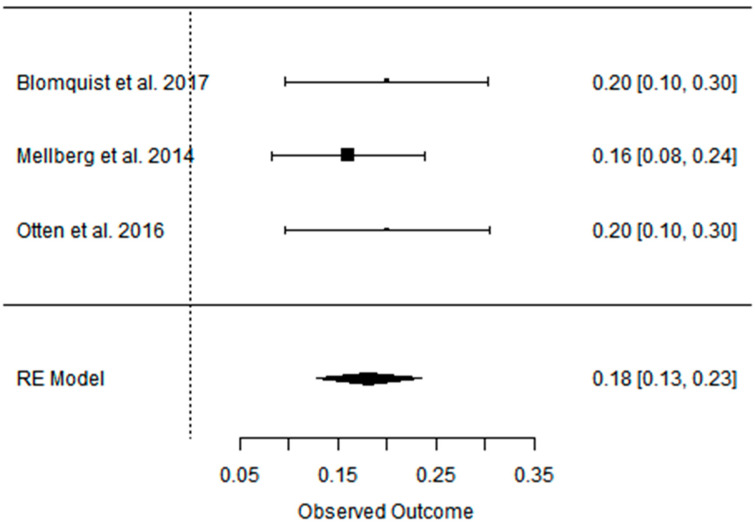
Forest plot of the effect of a PD on HDL-C in the long term (weighted mean difference).

**Figure A31:**
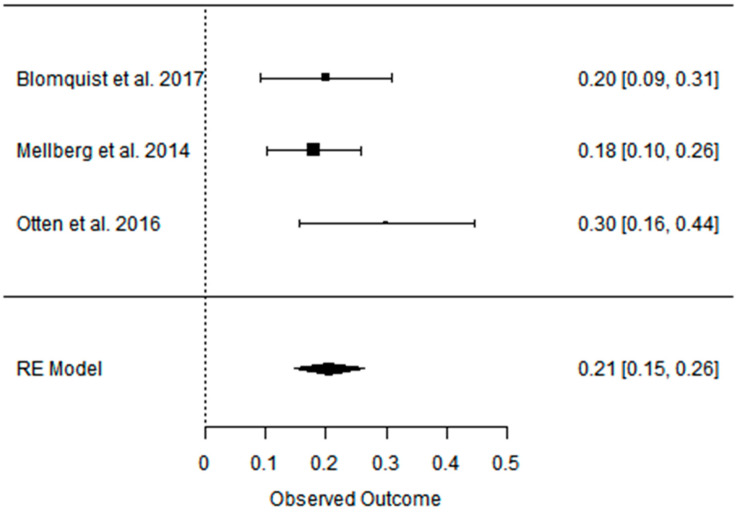
Forest plot of the effect of a CD on HDL-C in the long term (weighted mean difference).

**Figure A32:**
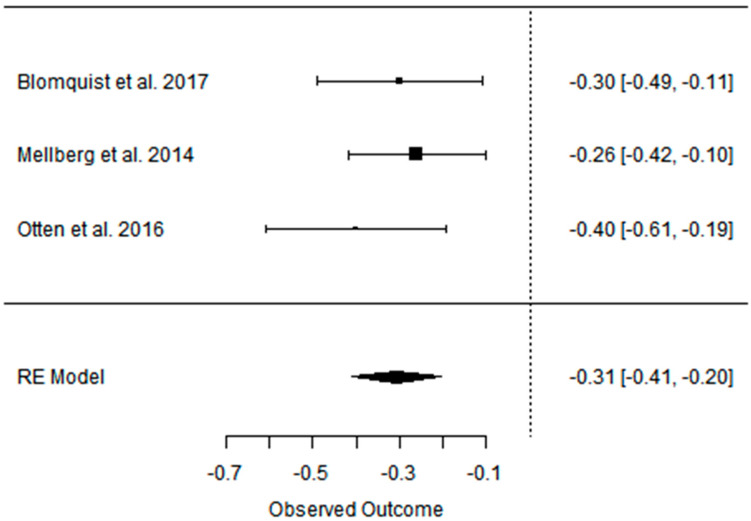
Forest plot of the effect of a PD on LDL-C in the long term (weighted mean difference).

**Figure A33:**
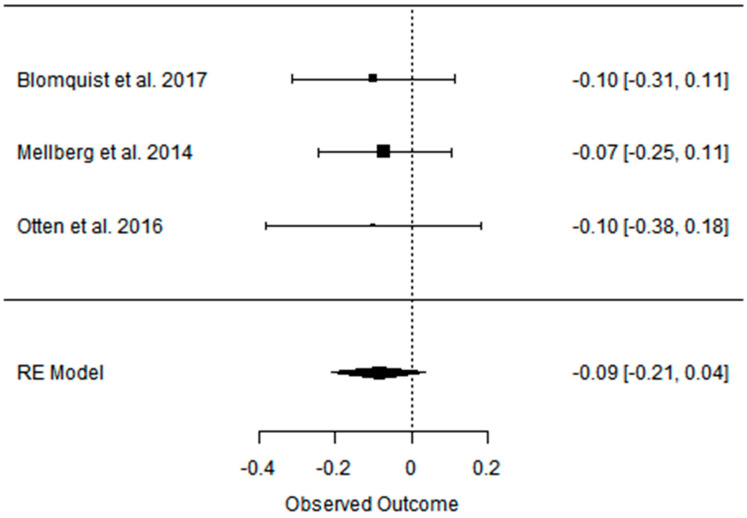
Forest plot of the effect of a CD on LDL-C in the long term (weighted mean difference).

All studies where the long-term impact of the Paleo diet on lipid profile metabolism indicators was investigated are PD vs. CD studies. Thus, in what follows, we describe only the impact of the PD and CD, without differentiating the type of study. The long-term impact of the Paleo diet on TC within the investigated studies (four) was statistically significant at the 0.05 level, being on average −0.23 mg/dL (95% confidence bands: −0.08; −0.38) [41,45,47,49] (Figure A26). However, the impact of the control diets in these studies was not statistically significant, with a reverse sign, being on average 0.01 mg/dL (95% confidence bands: 0.05; −0.03) [41,45,47,49] (Figure A27). The long-term impact of the Paleo diet on TG within the investigated studies (four) was statistically significant at the 0.001 level, being on average −0.23 mg/dL (95% confidence bands: −0.14; −0.30) [41,45,47,49] (Figure A28). The impact of the control diets in these studies (four) was statistically significant at the 0.05 significance level, being on average −0.08 mg/dL (95% confidence bands: −0.00; −0.15) [41,45,47,49] (Figure A29). The long-term impact of the Paleo diet on HDL-C within the investigated studies (three) was statistically significant at the 0.001 level, being on average 0.18 mg/dL (95% confidence bands: 0.13; 0.24) [41,47,49] (Figure A30). Simultaneously, the impact of the control diets in these studies (three) was also statistically significant at the 0.001 level, being on average 0.21 mg/dL (95% confidence bands: 0.15; 0.26) [41,47,49] (Figure A31). The long-term impact of the Paleo diet on LDL-C within the investigated studies (three) was statistically significant at the 0.001 level, being on average −0.31 mg/dL (95% confidence bands: −0.20; −0.41) [41,47,49] (Figure A32). However, the long-term impact of the control diets in these studies (three) was not statistically significant [41,47,49] (Figure A33) (Table 5).

### 3.4. Effect of the Paleolithic Diet on Blood Pressure (Systolic Blood Pressure, Diastolic Blood Pressure, and Heart Rate) in the Short (up to 6 Months) and Long (over 6 Months) Term

**Figure A34:**
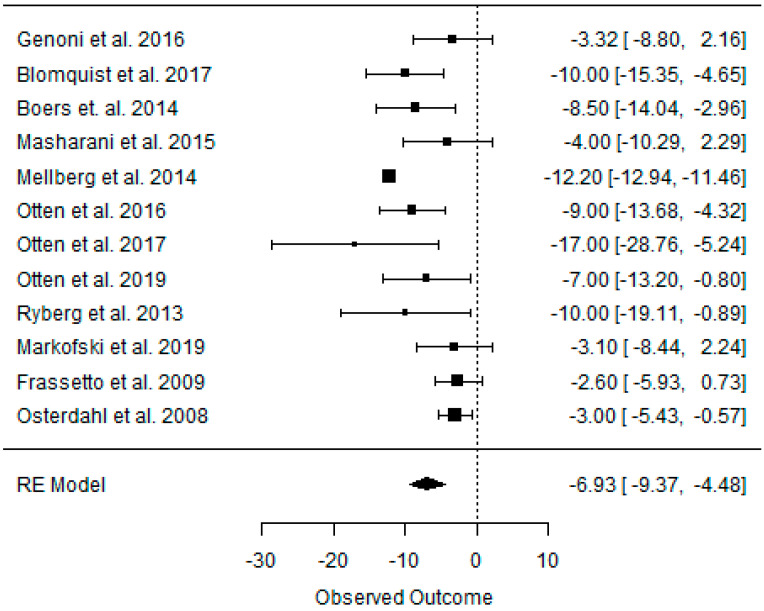
Forest plot of the effect of a PDs on SBP in the short term (weighted mean difference).

**Figure A35:**
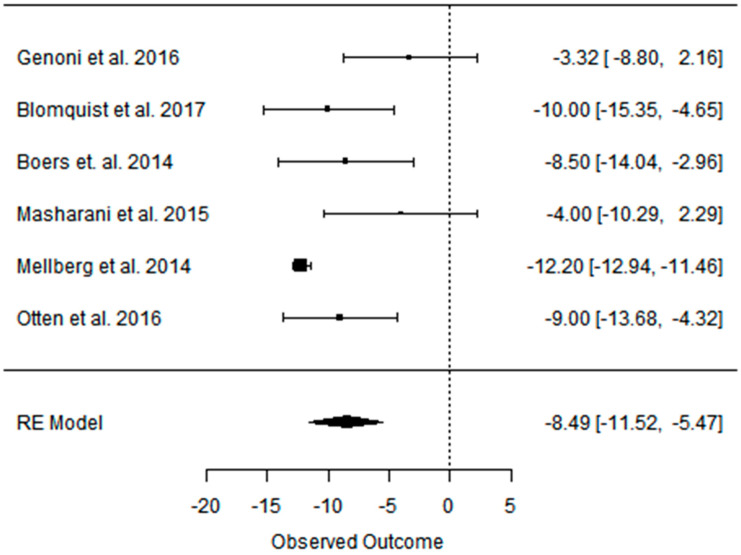
Forest plot of the effect of PD on SBP in the short term (weighted mean difference).

**Figure A36:**
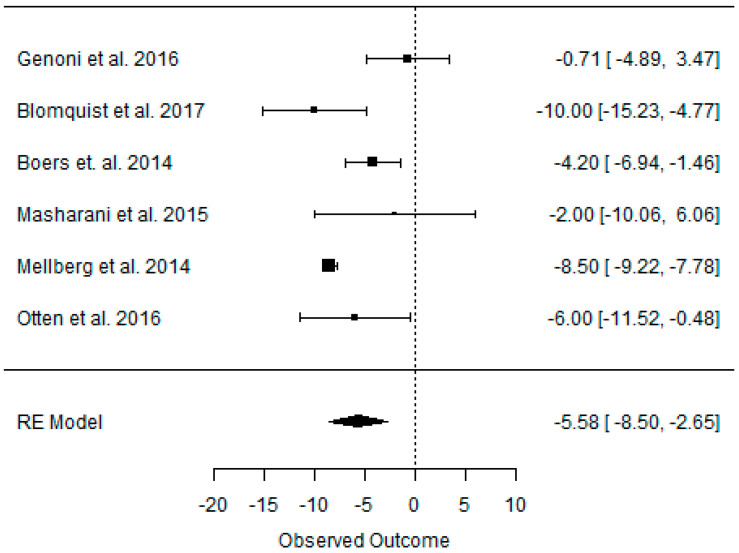
Forest plot of the effect of a CD on SBP in the short term (weighted mean difference).

**Figure A37:**
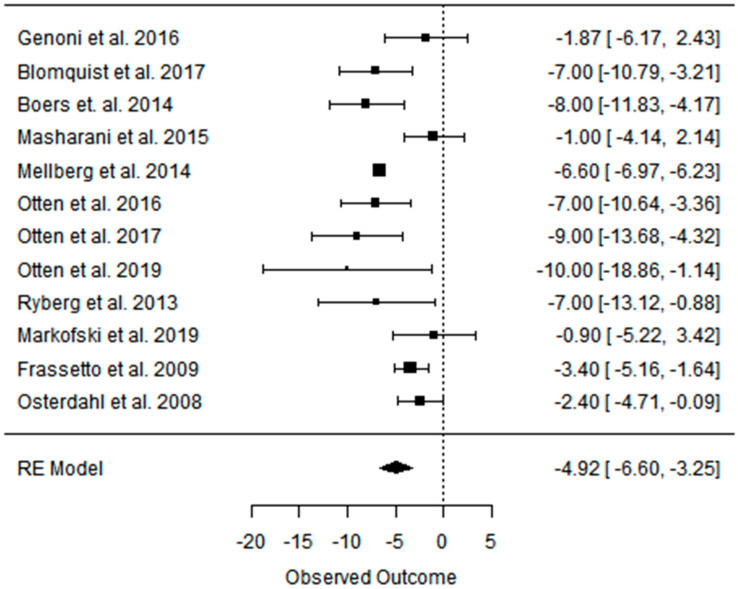
Forest plot of the effect of a PDs on DBP in the short term (weighted mean difference).

**Figure A38:**
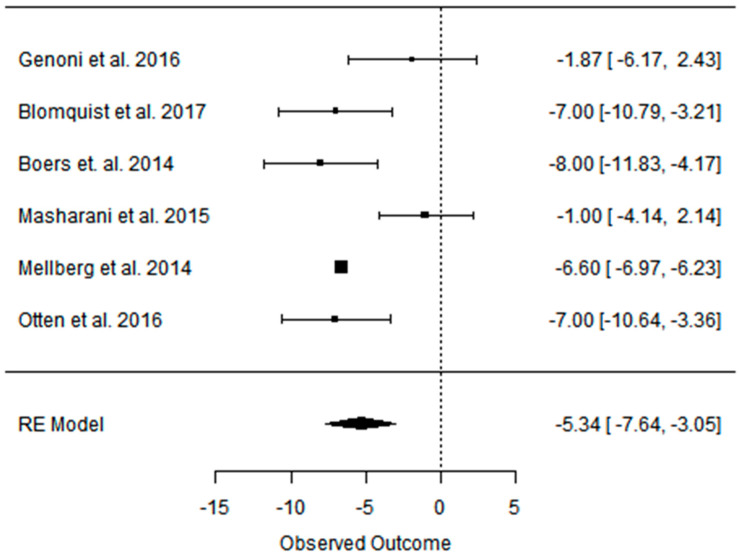
Forest plot of the effect of PD on DBP in the short term (weighted mean difference).

**Figure A39:**
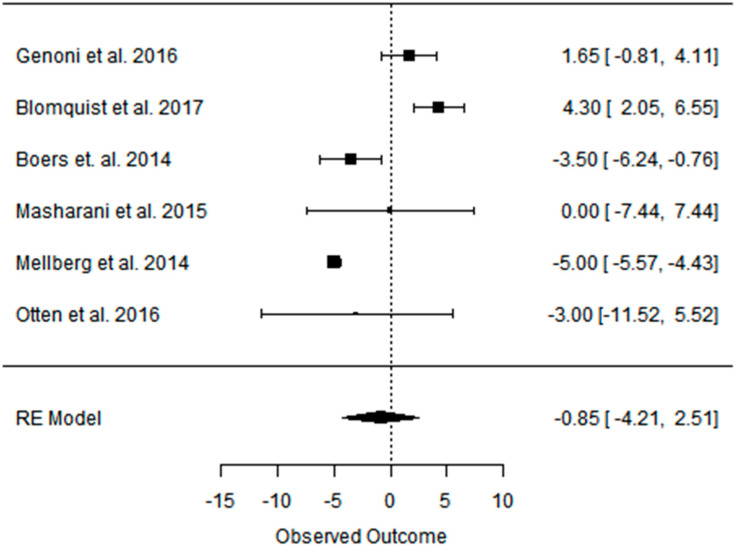
Forest plot of the effect of a CD on DBP in the short term (weighted mean difference).

The impact of the Paleo diet on SBP in all independently investigated studies (12) with this indicator was on average −6.9 mmHg (95% confidence bands: −4.5; −9.4) and was statistically significant at the 0.001 confidence level [18,37,41,42,44,47,49,52,53,54,56,71] (Figure A34). We observed a visibly higher effect (−8.5 mmHg, 95% confidence bands: −5.5; −11.5) in studies on PD vs. CD (six studies) [41,47,49,52,53,54] (Figure A35). Simultaneously, the control diets in these studies (six) also significantly reduced the SBP of the participants, albeit the average effect was substantially smaller (−5.6 mmHg with 95% confidence bands: −2.7; −8.5) [41,47,49,52,53,54] (Figure A36). The impact of the Paleo diet on DBP in all independently investigated studies (12) with this indicator was on average −4.9 mmHg (95% confidence bands: −3.2; −6.6) and was statistically significant at the 0.001 confidence level [18,37,41,42,44,47,49,52,53,54,56,71] (Figure A37). We observed a slightly higher effect (−5.3 mmHg, 95% confidence bands: −3.0; −7.6) in studies on PD vs. CD (six studies) [41,47,49,52,53,54] (Figure A38). However, the control diets in these studies (six) on average reduced the DBP of the participants only by 0.8 mmHg, which is not statistically significant [41,47,49,52,53,54] (Figure A39). The impact of the Paleo diet on HR in all independently investigated studies (four) with this indicator was on average −3 bpm and was not statistically significant [37,42,44,49]. Only in one study was the impact of PD vs. CD on heart rate investigated [49]. The impacts of the PD and CD was statistically significant at the 0.001 level, and that of the CD was greater (3.2 bpm vs. 2.2 bpm) [49] (Table 6).

**Figure A40:**
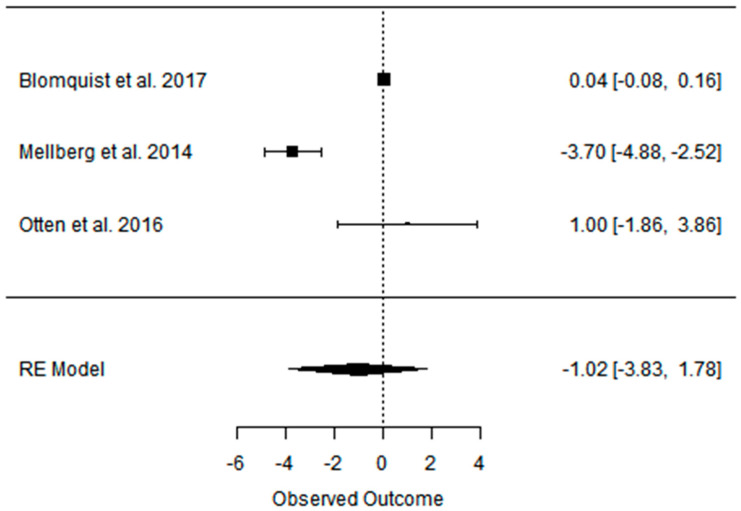
Forest plot of the effect of a PD on SBP in the long term (weighted mean difference).

**Figure A41:**
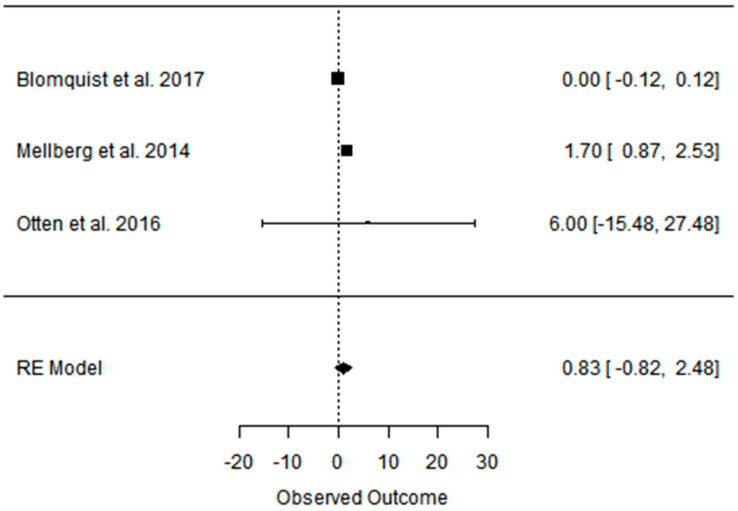
Forest plot of the effect of a CD on SBP in the long term (weighted mean difference).

**Figure A42:**
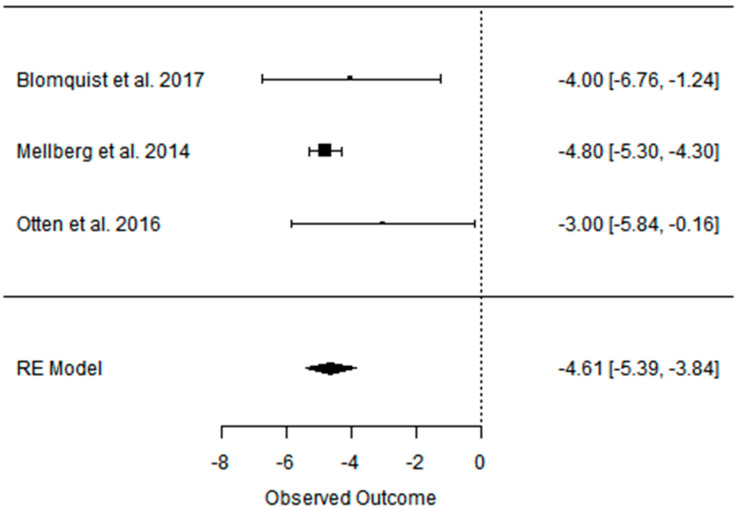
Forest plot of the effect of a PD on DBP in the long term (weighted mean difference).

**Figure A43:**
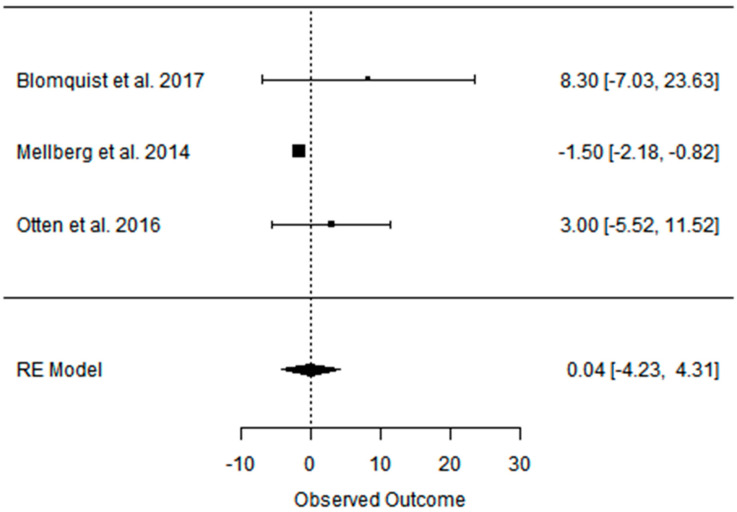
Forest plot of the effect of a CD on DBP in the long term (weighted mean difference).

All studies where the long-term impact of the Paleo diet on blood pressure indicators was investigated are PD vs. CD studies. Thus, in what follows, we describe only the impact of the PD and CD, without differentiating the type of study. The long-term impact of the Paleo diet (three studies) on SBP was not statistically significant, being on average −1 mmHg [41,47,49] (Figure A40). Similarly, the long-term impact of the CDs in these studies (three) was statistically not significant but was on average positive (0.8 mmHg) [41,47,49] (Figure A41). The long-term impact of PD (three) on DBP was on average −4.6 mmHg. (95% confidence bands: −3.8; −5.4) and was statistically significant at the 0.001 confidence level [41,47,49] (Figure A42). However, the long-term impact of the CDs in these studies (three) was statistically not significant, being on average nearly 0 [41,47,49] (Figure A43) (Table 7).

### 3.5. Effect of the Paleolithic Diet on Carbohydrates Metabolism (Fasting Plasma Glucose, Fasting Plasma Insulin, HOMA-IR, and HbA1c) in the Short (up to 6 Months) and Long (over 6 Months) Term

**Figure A44:**
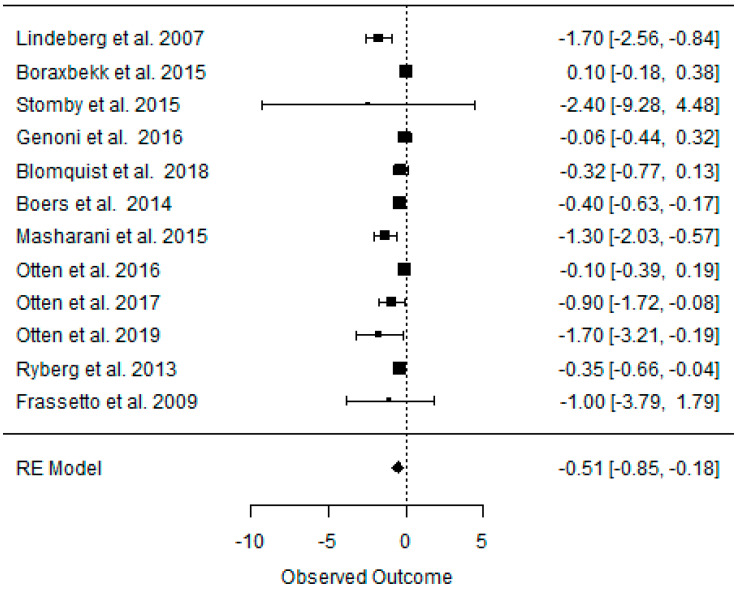
Forest plot of the effect of a PDs on fP glucose (mmol/L) in the short term (weighted mean difference).

**Figure A45:**
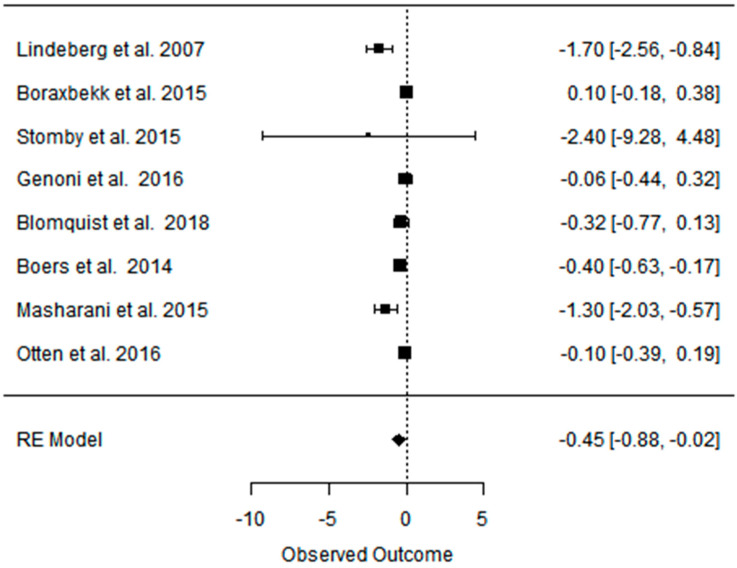
Forest plot of the effect of PD on fP glucose (mmol/L) in the short term (weighted mean difference).

**Figure A46:**
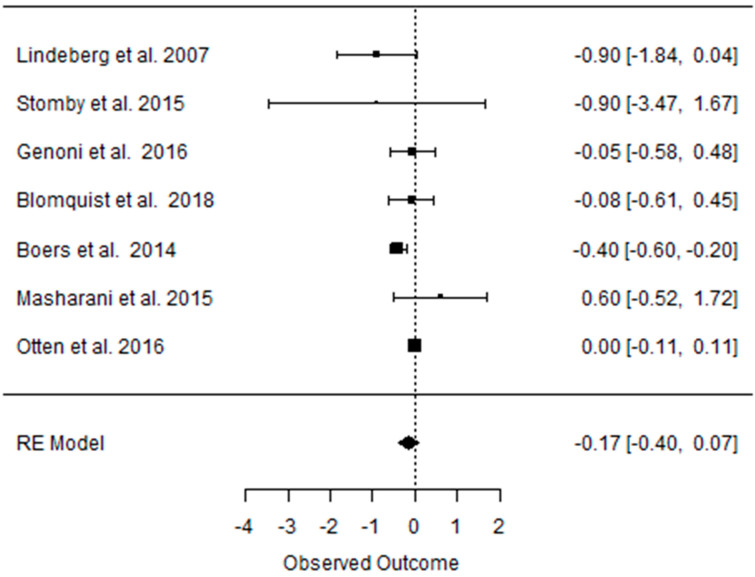
Forest plot of the effect of a CD on fP glucose (mmol/L) in the short term (weighted mean difference).

**Figure A47:**
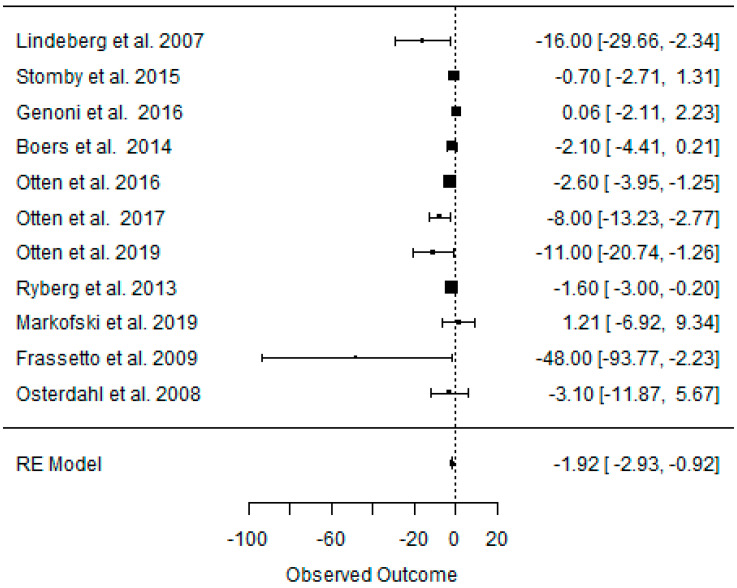
Forest plot of the effect of a PDs on fP insulin (mmol/L) in the short term (weighted mean difference).

**Figure A48:**
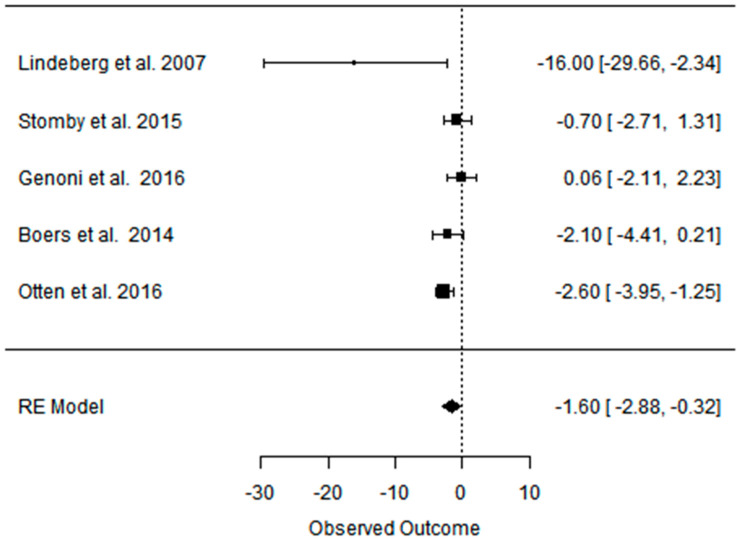
Forest plot of the effect of PD on fP insulin (mmol/L) in the short term (weighted mean difference).

**Figure A49:**
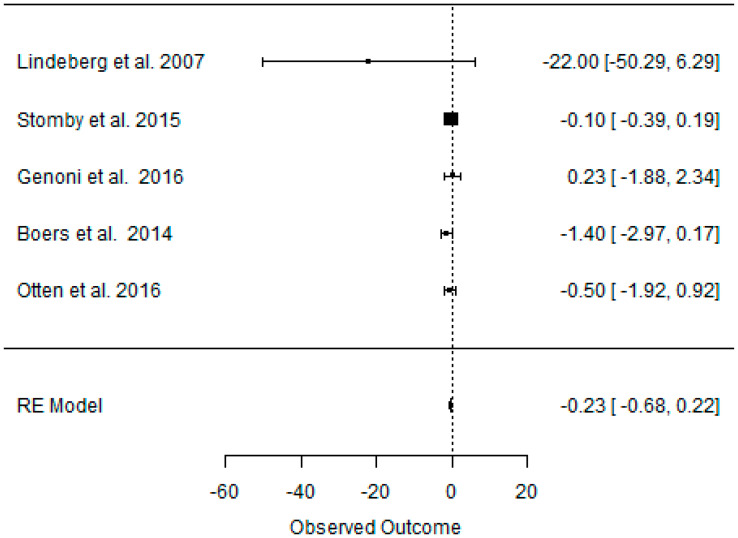
Forest plot of the effect of a CD on fP insulin (mmol/L) in the short term (weighted mean difference).

**Figure A50:**
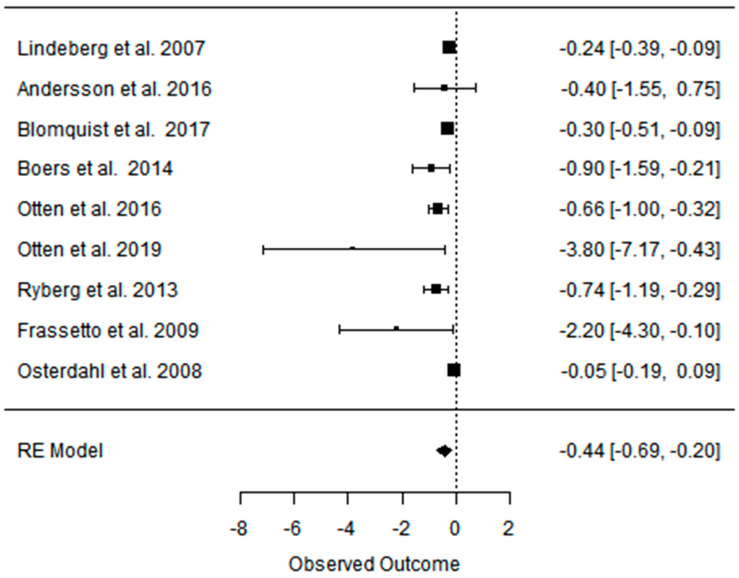
Forest plot of the effect of a PDs on HOMA-IR in the short term (weighted mean difference).

**Figure A51:**
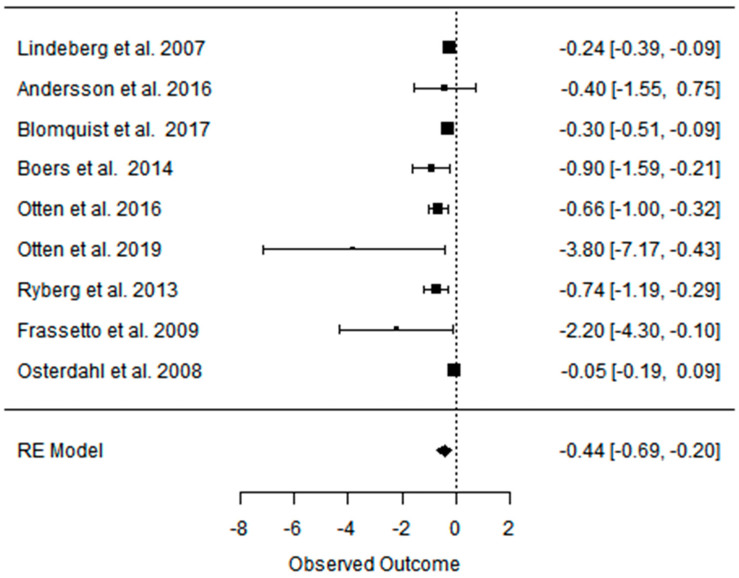
Forest plot of the effect of PD on HOMA-IR in the short term (weighted mean difference).

**Figure A52:**
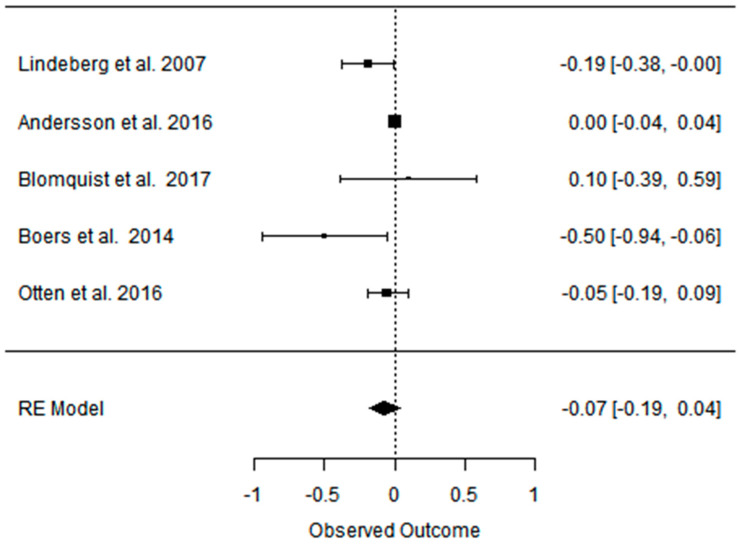
Forest plot of the effect of a CD on HOMA-IR in the short term (weighted mean difference).

The impact of the Paleo diet on fP glucose in all independently investigated studies (12) with this indicator was on average −0.51 mmol/L. (95% confidence bands: −0.2; −0.8) and was statistically significant at the 0.01 level [18,37,41,42,44,47,49,52,53,54,56,71] (Figure A44). We observed a slightly lower effect, which was statistically significant at the 0.05 level (on average −0.45 mmol/L, 95% confidence bands: −0.0; −0.9), in studies on PD vs. CD (eight studies) [28,40,41,46,48,52,53,54] (Figure A45). The CDs in these studies (seven) had no statistically significant impact on fP glucose (mmol/L), with an average lower impact (0.17 mmol/L) [28,40,41,46,48,52,53,54] (Figure A46). The impact of the Paleo diet on fP insulin in all independently investigated studies (11) with this indicator was on average −1.9 mmol/L. (95% confidence bands: −0.9; −2.9) and was statistically significant at the 0.001 level [18,28,37,41,42,44,46,53,54,56,71] (Figure A47). We observed a slightly lower effect, which was also statistically significant at the 0.01 level (on average −1.6 mmol/L, 95% confidence bands: −0.3; −2.9), in studies on PD vs. CD (five studies) [28,41,46,53,54] (Figure A48). However, the control diets in these studies (five) had no statistically significant impact on fP insulin [28,41,46,53,54] (Figure A49). The impact of the Paleo diet on HOMA-IR in all independently investigated studies (nine) with this indicator was on average −0.4. (95% confidence bands: −0.2; −0.7) and was statistically significant at the 0.001 level [18,28,37,41,44,45,47,53,56] (Figure A50). We observed a slightly lower effect, which was also statistically significant at the 0.001 level (on average −0.4 mmol/L, 95% confidence bands: −0.2; −0.7), in studies on PD vs. CD (five studies) [28,41,45,47,53] (Figure A51). However, the CDs in these studies (five) had no statistically significant impact on HOMA-IR [28,41,45,47,53] (Figure A52). The impact of the Paleo diet on HbA1c in all independently investigated studies (three) with this indicator was on average −0.4% (95% confidence bands: 0.0; −0.8) and was statistically significant at the 0.1 level [28,42,52]. We observed a visibly lower effect, which was statistically significant at the 0.05 level (on average −0.2%, 95% confidence bands: −0.0; −0.3), in studies on PD vs. CD (two studies) [28,52]. The CDs in these studies (two) also had a statistically significant impact on HbA1c at the 0.1 significance level but was visibly lower (on average −0.1%, 95% confidence bands: 0; −0.3) [28,52] (Table 8).

**Figure A53:**
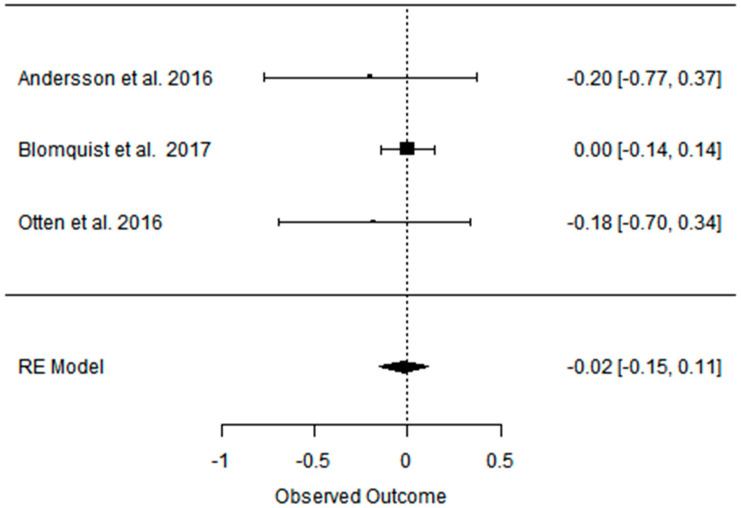
Forest plot of the effect of a PD on HOMA-IR in the long term (weighted mean difference).

**Figure A54:**
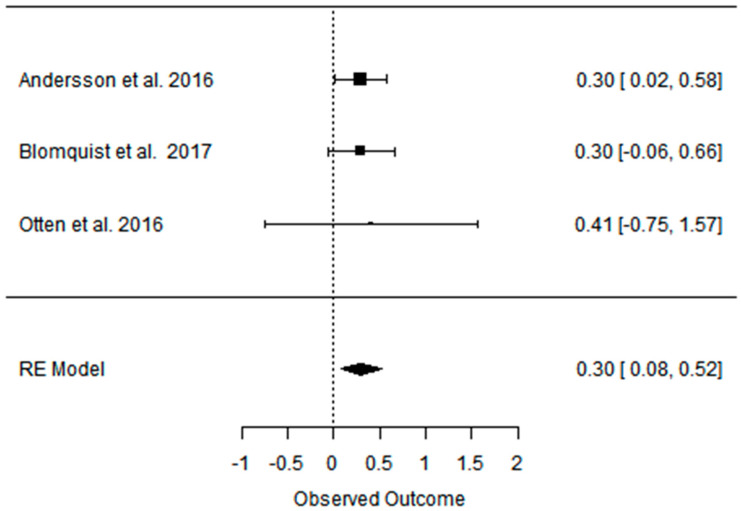
Forest plot of the effect of a CD on HOMA-IR in the long term (weighted mean difference).

All studies where the long-term impact of PD on carbohydrate metabolism indicators was investigated are PD vs. CD studies. Thus, in what follows, we describe only the impact of the PD and CD, without differentiating the type of study. The long-term impact of the Paleo diet on fP glucose within the investigated studies (two) was not statistically significant [41,46]. Similarly, the impact of the control diets in these studies (two) was statistically not significant, being near 0 on average [41,46]. The long-term impact of the Paleo diet (two studies) on fP insulin was not statistically significant [41,46]. The long-term impact of the control diets in these studies (two) was similar [41,46]. The long-term impact of the Paleo diet on HOMA-IR within the investigated studies (three) was not statistically significant [41,45,47] (Figure A53). However, the impact of control diets in these studies (three) was statistically significant at the 0.01 level, being on average 0.3 (95% confidence bands: 0.09; 0.5) [41,45,47] (Figure A54) (Table 9).

### 3.6. Effect of the Paleolithic Diet on Physical Capacity (Maximum Oxygen Uptake and Maximum Workload)

The impact of PD on VO_2_max (mL/kg/min) in all independently investigated studies (three) with this indicator without exercise increased on average 2.1 mL/kg/min (95% confidence bands: 1.3; 2.8) and was statistically significant at the 0.001 level [42,44,55]. We observed a slightly lower effect of the Paleo diet without exercise, which was also statistically significant at the 0.001 level (on average 1.9 mL/kg/min, 95% confidence bands: 1.1; 2.7), in studies on PD vs. PD + EX (two studies) [42,44]. The impact of the Paleo diet with exercise was much higher and statistically significant at the 0.001 level, reaching 3.5 mL/kg/min (95% confidence bands: 2.0; 4.9) [42,44]. Furthermore, we found that maximum workload (W max) was statistically significant (at the 0.05 level) only for PD + EX in 44 studies [44] (Table 10).

## 4. Discussion

The present meta-analysis evaluated randomized clinical trials (RCTs). The population groups differed significantly in clinical characteristics, since individuals with ischemic heart disease were either diabetic, were hypercholesterolemic, were obese or overweight, had metabolic syndrome, or were healthy. We also included a study where the Paleo diet was combined with physical activity in healthy or unhealthy volunteers (i.e., T2DM). Our meta-analysis confirms that even the short-term use of the Paleolithic diet improves health status indicators, metabolic biomarkers, and body composition (normalization of blood pressure, improvement in the lipid profile, a lowering of total cholesterol, TG, and LDL, increases in HDL, increased glucose tolerance and insulin sensitivity, losses in body and fat mass, lower waist circumference, and decreases in BMI) in obese people, suffering from type 2 diabetes and metabolic syndrome, and healthy people to a greater or similar extent compared to other healthy diets [18,28,37,39,40,41,42,43,44,45,46,47,48,49,50,51,52,53,54,55,56,71]. These changes improved health, especially in obese patients (body mass reduction), patients with diabetes (improved carbohydrate metabolism) or cardiovascular disease (normalization of blood pressure), and women with metabolic syndrome (fasting glucose, insulin sensitivity, abdominal obesity, lipid profile, and blood pressure all improved). Conclusions from the influence of this PD on other markers (i.e., net acid excretion, CRP, selected hormones, e.g., leptin and cortisol, and indicators analyzed in multiple sclerosis and other diseases, e.g., acne) [29,72,73,74] and the long-term use of PD must be interpreted with caution, as they concern single or several studies.

In our meta-analysis, we found that both Paleo diets and a variety of alternative healthy diets (control diets) caused a statistically significant reduction in BM, BMI, WC, and FM, both in the short and long term. For many indicators, such as body mass, BMI, or waist circumference, the impact of the Paleo diet was stronger than the impact of the control diet. We also noticed statistically significant differences in FM in PD (not in CD) in the short term. The only indicator on which the impact on both diets was inconclusive is LBM, on which the impact of both diets in the short term is neither statistically positive nor negative, while the long-term impact was investigated only in 41 (not included in meta-analysis) studies. Regarding the strategy connecting PD and physical activity, Otten et al. [42] observed decreased fat mass, body mass, and waist circumference in both the PD and PD-EX groups, without differences between intervention groups. Male participants decreased their waist circumference more in the PD group compared to the PD-EX group. Males in the PD-EX group retained more lean mass than males in the PD group [42]. The previous meta-analysis conducted by Ghaedi et al. [75] indicated that the Paleolithic diet (eight RCTs) could significantly decrease anthropometric indexes, including BM, WC, BMI, and FM %. These results were also confirmed by Manheimer [76], who pointed out that Paleolithic nutrition was more effective in reducing body weight in comparison to the control diet [76]. Menezes [17] in their meta-analysis (11 RCTs) found that PD may assist in controlling BM, BMI, and waist circumference. The summary of the effect showed a loss −3.52 kg in PD [17]. It should be noted that increased satiety has been reported in Paleo-type diets compared with other types of diets in a few studies [28,77]. It is worth emphasizing that there are few studies reporting a significant decrease in body weight, fat mass, and waist circumference in the short term (after 6 months) that is not sustained in the long term (after 24 months) in a Palaeolithic-type diet compared to a reference diet in a control group [45,46,49]. Our meta-analysis confirms the greater effect of changes in the shorter period of diet application than in the longer period. In the majority of the studies included in the meta-analysis (exceptions being those by Masharani et al. [52], Boers et al. [53], and Frassetto et al. [18]), the ad libitum diet regimen was introduced both in the PD and CD groups. The Paleolithic diet, rich in complete protein and fiber, can actually be characterized by a high degree of satiety [72,78], which could have resulted in a slightly greater reduction effect in PD. Moreover, in terms of the ad libitum model, energy intake was lower than the energy demand in both groups, and comparing the energy supply in both research groups, it was lower in the groups with PD in juxtaposition with CD [28,43]. It can therefore be concluded that the Paleolithic diet can be an effective reduction diet with a shorter duration of use compared to other healthy diets maintaining the ad libitum strategy. Similarly, in terms of particular components of the body composition, a more significant reduction effect was noted in the groups with PD or PD-EX interventions. Short observations (up to 6 months) showed a more notable adipose tissue loss in kg and %. Given the lesser effect in terms of longer interventions, perhaps the regimen associated with maintaining the Paleolithic diet (abandoning products that are the majority in the “Western” diet, i.e., highly processed foods, milk, cereal, and dairy products) is difficult to sustain over the long term. Thus, the lack of difference in measurements in the end could be due to the fact that, over time, the participants allowed themselves to make exceptions in the diet [45,46,49]. The above-mentioned changes were observed in obese women in the postmenopausal stage, people suffering from type 2 diabetes (not treated with insulin) and ischemic heart disease, and healthy people conducting physical activity on purpose. The reduction in energy intake is perhaps the explanation for the good influence of the Paleo diet on weight and waist circumference [28,37,39,40,42,44,47,48,51,52,54,55,56]. Additionally, a higher fruit consumption while on the Paleo diet is associated with a significant reduction in waist circumference in terms of people with diabetes [28]. No changes in the body weight of the participants were reported by Frassetto et al. [18]; in that case, the weight loss was not planned (the normoenergetic diet). It is worth pointing out that other LCDs that are rich in protein and/or fat also cause weight loss [79,80,81,82].

In our paper, overall, the Paleolithic diet and various healthy diets caused decreases in TC, LDL-C, and TG, albeit the impact of the Paleo diet was stronger. Among long-term studies, only the Paleo diet caused a statistically significant decline in TC and LDL-C. In PD and CD, we noticed a similar significant increase in HDL-C. The same results were found by Manheimer et al. [76], but they also noted beneficial changes in HDL-C, but this did not reach a significant level; however, improvements in metabolic syndrome components after the consumption of a Paleolithic nutritional pattern were observed [76]. In Ghaedi et al.’s meta-analysis [75], PD resulted in reduced TC, LDL-C, and TGs and elevated circulating concentrations of HDL-C. However, similar to Manheimer’s papers, according to the sensitivity analysis, Ghaedi et al. [75] found that the overall effects of a PD on the lipid profile were sensitive to the removal of some studies and to the correlation coefficients; thus, the effect of a PD in this field must be interpreted with caution [75]. Nevertheless, all meta-analyses, including ours, emphasize the beneficial effect of diet on the lipid profile, especially in obese people, those suffering from metabolic diseases, and healthy people [18,37,39,40,41,42,43,44,45,46,47,48,49,50,51,52,53,54,55,56]. Frassetto et al. [18] noticed that even short-term consumption of a Paleolithic type diet improves lipid profiles—just after 10 days, total cholesterol decreased by −16%, LDL-C decreased by −22%, and triglycerides decreased by −35%. No significant change occurred in HDL-C in nonobese and sedentary groups [18]. Paleolithic nutrition offers a promising potential for the nutritional management of hyperlipidemia in adults whose lipid profiles have not improved after following more traditional heart-healthy dietary recommendations [39]. Smith et al. demonstrated that an ad libitum, unrestricted PD was significantly deleterious to blood lipid profiles in healthy subjects participating in a CrossFit-based training program (high-intensity). Subjects with optimal initial blood lipid values demonstrated the greatest increase in LDL, TC, and n-HDL values, along with the greatest decline in HDL-C values, following a 10-week Paleo diet intervention. Despite the concurrent improvements in aerobic capacity and body composition noted in these subjects, the PD may have negated the positive effects of exercise on blood lipids in Smith’s studies [55]. PD in overweight or obese postmenopausal women had a significant and persistent effect on liver fat and differed significantly from a conventional LFD at 6 months. This difference may not be due to greater body weight reduction but to a difference in food quality, for example, a higher content of mono- and polyunsaturated fatty acids in the PD, due to the authors [41].

Statistically significant impacts of Paleolithic and various healthy diets on blood pressure were observed mainly in the short term. Overall, the Paleo diet had a stronger impact on both SBP and DBP. In the long term, the only significant impact on DBP was seen in the Paleo diet. The other, pooled analysis also showed that a PD led to significant reductions in both SBP and DBP. However, according to a sensitivity analysis, Ghaedi et al. [75] found that the overall effects of a PD on SBP concentrations were sensitive to the removal of some studies and to the correlation coefficients; thus, the effect of a PD in this field must be interpreted with caution as well. [75]. In the studies we took into account in our meta-analysis, a decrease in both systolic and diastolic blood pressure was observed in subjects with metabolic syndrome, those overweight, those with a sedentary lifestyle, and those with type 2 diabetes [18,37,41,42,44,47,49,52,53,54,56,71]. Even the short-term consumption of a PD improves blood pressure [18,53,56,71]. In other studies of longer duration (up to 12 weeks—still short term), changes in single indices related to blood pressure were observed in healthy subjects (a decrease in systolic blood pressure) [56], obese postmenopausal women, and people with type 2 diabetes not treated with insulin (a decrease in diastolic blood pressure) [37,41,78]. A PD can be effective in reducing blood pressure in hypertensive people [79] because of the high intake of fruit and vegetables, and this dietary pattern is rich in potassium content [83]. Most researchers conclude that, in the prevention of hypertension, an appropriate potassium/sodium ratio, which is characteristic of the Paleo diet, is important [18,23,83].

In our meta-analysis, PD caused a statistically significant decrease in fP glucose, fP insulin, HOMA-IR, and HbA1c in the short term—contrary to various CDs, which caused only a statistically significant decrease in HbA1c. In the long term, the Paleo diet caused no statistically significant changes in all carbohydrate metabolism indicators. Simultaneously, the various CDs in the long term similarly caused no statistically significant impact on fP glucose and fP insulin, while its impact on HOMA-IR was statistically significant. In a recent meta-analysis, Jamka et al. [84] presented that the PD (four RTCs) did not differ from other types of diets commonly perceived as healthy regarding its effect on fasting glucose and insulin concentrations, AUC 0–120 glucose and AUC 0–120 insulin levels, HbA1c values, and the HOMA-IR index. A decrease in fP glucose concentrations in the Paleolithic group was observed in the end of the intervention period in most of the studies included in this systematic review. The Paleolithic diet did not differ from other types of diets commonly perceived as healthy with regard to effects on glucose and insulin homeostasis in subjects with altered glucose metabolism. Several, albeit not all, studies have suggested that the consumption of the Paleolithic diet might improve glucose tolerance, decrease insulin secretion, and increase insulin sensitivity [84]. Similar results were found in another meta-analysis, showing that beneficial changes of fP glucose values did not reach significance, but the analysis suggested short-term improvements in metabolic syndrome components after PD consumption [76]. Taking into account carbohydrate metabolism, an improvement in glucose tolerance and/or insulin sensitivity was observed in overweight people and patients with type 2 diabetes [28,37,48,52]. The nutritional composition of a Paleo diet—high-fiber content, high antioxidants, high mono- and polyunsaturated fats, low sodium, and high potassium—may be particularly beneficial in patients with T2DM, even if they are on medicine to control glucose levels, BP, and lipids [52]. There was a greater positive effect of the Paleo diet on carbohydrate metabolism compared to the Mediterranean diet [28] and the ADA diet [52]. Moreover, the Paleo diet had the same effect on satiety as the Mediterranean diet, but with a lower caloric intake during the day (ad libitum intake in both groups). There was a greater decrease in glucose (but not insulin—the same as noted by Lindeberg et al. [28]) in diabetics following the Paleo diet compared to the usual diet, and it is worth noting that, in the Paleo group, the subjects consumed up to 1 kg of fruit per day (both groups reported energy intake, and the height of the glycemic index was at a similar level) [28,51]. Conventionally, the glycemic index is lower in the Paleolithic nutrition model (GI = 50) compared to the diabetic diet (GI = 55) [78]. According to Frassetto et al., even short-term consumption of a Paleolithic type diet improves glucose tolerance, decreases insulin secretion, increases insulin sensitivity—without weight loss [18]. In addition, studies have shown that the low carbohydrate/protein ratio commonly associated with the Paleo diet improves glycemic control and body composition (PD may improve glucose tolerance independently of positive changes in WC) [18,28,55]. Similarly, in one intervention, the energy intake and macronutrients (Paleo vs. control, based on the recommendations of the ADA) was the same in both groups. In addition, the energy content of both diets was set at a level that prevents weight loss. In many studies, the Paleo diet was characterized with lower energy and carbohydrate content than the control diets; thus, the question arises: Is the beneficial effect of the Paleo diet due to the elimination of grain and dairy products or merely a reduced caloric intake? Both groups improved fasting glucose and lipid profile, but the effect of the Paleo diet was more favorable. Additionally, in the Paleo group, an improvement in insulin sensitivity was noted in patients with the highest insulin resistance. No such effect was noted in the ADA group. Despite a number of advantages, the discussed study also has disadvantages: the dietary fiber in the ADA group was much lower than in the Paleo group (12 g vs. 35 g/d), which could have influenced the results [52]. The larger improvement of glucose tolerance in the Paleolithic group was independent of energy intake and macronutrient composition, which suggests that avoiding Western foods is more important than counting calories, fat, carbohydrates, or protein. The study adds to the notion that healthy diets based on whole-grain cereals and low-fat dairy products are only the second-best choice in the prevention and treatment of type 2 diabetes [28]. An improvement in insulin sensitivity and a decrease in the percentage of HbA1c were also observed in Otten’s studies, in which DP was used in combination with resistance training. Lower energy consumption was observed in people who undertook physical activity [42].

It is doubtful whether the positive effect of the ancestral diet on health is caused by the elimination of highly processed foods/specific products (cereals, milk and dairy products, and legume seeds) from a diet, e.g., that is rich in some anti-nutritional substances, has a high content of vegetables and fruits, or is energy-deficient, which results in weight loss. It has been known for a long time that the correct body structure affects health. It is worth noting that, in the cited scientific studies, the ad libitum model of consumption was mostly used both in the groups on a Paleo diet and in control groups characterized by a different diet, usually therapeutic (e.g., “diabetic”). In addition, in the interventions with the assumption of caloric intake dictated by the feeling of satiety (ad libitum) [28,37,38,39,40,41,42,43,44,45,46,47,48,49,50,51,52,53,54,55,56,71], a lower energy intake was observed in the PD groups [28,41]. In some of the studies, the participants did not have strictly imposed proportions of macronutrients or proportions of animal and plant products. They reached for the particular products of their preferences. There are few scientific reports in which the normoenergetic model of nutrition is proposed [18,52,53]. However, the Paleo diet with the ad libitum nature of consumption cannot be completely eliminated, because even short-term use of PD with the isocaloric nature has been associated with a normalization of blood pressure, glucose tolerance, decreased insulin, an increase in insulin sensitivity, and lipid profile improvement [18], without weight loss in healthy people, leading to a sedentary lifestyle. A similar effect was noted in the group with at least two factors of the metabolic syndrome [53]. In the studies by Masharani et al. [52] in people with type 2 diabetes, a positive effect was noted in the lipid profile (the PD group had statistically significant declines in TC, HDL-C, and LDL-C), carbohydrate metabolism (the PD group had greater benefits on glucose control and significant improvement in insulin sensitivity), and, interestingly, body composition (the average weight changes were similar in both groups without caloric restriction), while the pressure remained unchanged (the mean arterial pressure did not significantly change in any of the two groups) [52].

On the other hand, an umbrella review that assessed meta-analyzes of randomized controlled trials of various diets (including PD) effects on anthropometric parameters and cardiometabolic risk factors were recently published and criticized the scientific evidence on the effects of using this diet. The authors considered two metanalyses which we also discussed (Ghaedi [75] and Manheimer [76]) and as it turned out the evidence for paleolithic dietary patterns was graded as weak [85].

There are studies that include PD + EX which conclude that the Paleolithic diet is a powerful tool to improve body composition and metabolic balance, including insulin sensitivity, glycemic control, and leptin in individuals with type 2 diabetes. Supervised exercise training did not provide additional effects on these outcomes but preserved lean mass in men and increased cardiovascular fitness were observed [42].

We observed a positive impact of a Paleo diet with exercise on all investigated athletic performance. All results, due to a low number of studies, should be interpreted cautiously. Despite the low number of studies, we took them into account because they are the only studies that analyzed physical performance in unhealthy (obese or T2DM) or healthy but not active subjects on a daily basis. Maximum oxygen (L/min) uptake increased in the PD-EX group only in Otten et al.’s study [42]. Otten et al. [44] found that a PD with exercise improves VO_2_max in subjects with type 2 diabetes while participating in supervised exercise training. It is worth emphasizing, that the difference between PD and PD-EX is the effect of exercise training, not the effect of PD. Smith et al. [55] presented increased peak oxygen uptake. However, regarding the Paleo diet, despite concurrent improvements in aerobic capacity and body composition noted in these subjects, the Paleo diet may have negated the positive effects of exercise on blood lipids [55]. The long-term effects of this intervention and the prognostic value of our findings need to be addressed in larger prospective studies. In Popp’s study, the results suggest a MyPlate diet with both aerobic and resistance training, which, more so than PD recommendations with exercise, improves aerobic fitness [70].

Taking into account athlete health, it has been shown that the use of LCDs adversely affects the lipid profile and promotes atherosclerotic changes in athletes [86,87]. The LCD also shows positive changes in fasting blood TG, LDL-C, glucose, insulin, and HOMA-IR, but they were not significant in a group of basketball players [88]. There is also no negative impact of high-protein diets on kidney or liver function in athletes [89]. There is a lack of metabolic imbalances and adverse health effects as long as adequate amounts of energy and protein are provided [81,82].

Kang’s review indicates that a non-calorie-restricted KD provides minimal ergogenic benefits in normal-weight individuals including athletes but can be used for optimizing BM and body composition without compromising aerobic and anaerobic performance [89]. Studies conducted on a group of professional and inactive athletes showed weight loss without energy restrictions when using diets with lower carbohydrate content and increasing fat consumption (LCD/VLCD-KD); there was a loss of body weight—specifically, a decrease in fat mass while maintaining fat-free mass, which is extremely important in terms of practicing sports [79,90,91,92,93]. The benefits of the LCD were the reduction in body fat [59]. McSwiney [94] demonstrated that a short-term (seven days), ad libitum KD consumed by trained men produces a ~3% loss of body mass and marked changes in substrate utilization across a range of exercise intensities. Although such changes in substrate utilization may be considered to be ergogenic in some performance contexts (ultra-endurance) and generally reflect the outcomes of longer-term adherence to a KD, it remains to be confirmed whether a short-term KD confers a performance benefit.

Moreover, a KD with a caloric surplus combined with resistance training, in trained men, would have a positive effect on the reduction in body fat and would be profitable for the increase in fat-free body mass [95]. The question is: Would there be any fat-free mass gain/fat mass loss if the hypercaloric Paleo diet was combined with resistance training? From the point of view of athletes, strategies that allow them to effectively reduce body weight without compromising health, a loss of muscle mass, and exercise capacity are important. There are hitherto no studies assessing the impact of the Paleolithic diet on body composition components of competitive athletes. It is also worth noting that changing the consumption of macronutrients may affect body weight. Michalczyk et al. [87] showed results that confirm that the level of body fat does not depend on the amount of fat consumed in the diet, but on the amount and quality of carbohydrate consumption. Switching to a low-carbohydrates ketogenic diet (LCKD) reduces fluid retention and, in turn, contributes to weight loss. An LCKD changes the efficiency of metabolic pathways [90,96] and increases the oxidation of fatty acids [65,97]. It seems that the above mechanism does not have to occur when using a PD, where the intake of carbohydrates is much higher than in an LCKD, but lower than in the usual diet.

On the other hand, LCDs accelerate the feeling of fatigue and reduce concentration, decreasing the effectiveness of training [55,98,99]. Contemporarily, dietary guidelines for athletes emphasize a high carbohydrate intake. This is related to concerns about the impact of glycogen depletion on fatigue and adaptation [100,101], but the role of exogenous carbohydrates in this context has recently been questioned [102]. For example, there is no evidence that carbohydrates are required for mTORC-1 (mammalian target of rapamycin complex-1) signaling during muscle protein synthesis [103], when dietary protein intake is sufficient [102]. In addition, the metabolic shift induced by an LCKD towards fat oxidation (and glycogen sparing) may have a beneficial effect on ATP resynthesis during strength training [98]. In this context, it is worth analyzing diets that do not lead to ketosis [104] but are characterized by a lower carbohydrate content, e.g., the Paleo diet. It is not entirely clear whether a moderate-carbohydrate diet can provide ergogenic benefits for athletes that are characterized by a proper body weight and whose primary goal is to improve performance and not lose weight. The doubt concerns the fact that, for example, KDs may affect the loss of body weight and adipose tissue by enhancing the oxidation of fatty acids, and may also reduce glycogen resources, which may result in reduced athletic performance [89]. It remains unclear whether a similar benefit can be expected from the use of the normoenergetic Paleo diet by athletes. One certain advantage is the sufficient carbohydrate content (glycogen), but a disadvantage may be a lack of ketosis in the KD, enabling the use of fatty acids in energy processes.

For some sports, e.g., team sport athletes, a high intake of carbohydrates may not be necessary in a daily diet, due to less training and a shorter competition time; in this case, PD might be a good choice. However, there are disciplines, e.g., dynamic team sports, for which the consumption of carbohydrates is recommended before, during, and after competition or training [105]. The question is of whether in this case the amount of carbohydrates from the Paleo diet will be sufficient. A previous review and meta-analysis showed that, when comparing the endurance athlete’s ketogenic diet (EAKD) to a high carbohydrate diet, there are mixed findings for the effect of EAKD consumption on endurance performance. Bailey and Hennessy reported non-significant VO_2_max outcomes [62,92,104,106,107]. This review provides evidence that EAKD consumption produces mixed results, in terms of endurance performance, when compared to a high carbohydrate diet.

According to Bujko and Kowalski, a PD can have an ergogenic impact on the body of the athlete due to the high creatine content in the diet corresponding in terms of the amount of branched-chain amino acids (BCAAs). The Paleo diet is a diet rich in animal protein, which is the best source of BCAAs—valine, leucine, and isoleucine, which stimulate muscle growth and regeneration. Due to the alkalizing properties of fruit and vegetables, it can also have a normalizing effect on the acid–base balance, despite the high content of animal protein. The high content of vitamins and minerals and the low amount of anti-nutritional ingredients (including gluten, phytic acid, lectins, and saponins) in cereal products and legumes, important in terms of increased energy and nutrient requirements. Proper proportions of omega-6/omega-3 fatty acids in the diet (difficult to achieve in the long term) and the elimination of insulinotropic properties may be valuable as well [67]. It should be emphasized that there are no studies assessing the impact of the use of the Paleo diet on exercise capacity and the health conditions of professional athletes at present. The influence on exercise capacity (especially during weight reduction) in the case of the ad libitum strategy seems to be even more ambiguous. In such a situation (during ad libitum), there is a risk of a very low supply of energy, vitamins, and minerals. On the other hand, it seems to be relatively difficult to balance a diet for athletes with high energy expenditure (>4000 kcal), who want to apply the diet for reasons such as the potential ergogenic benefits, the elimination of highly processed foods or of gluten and/or lactose, or the reduction in carbohydrate intake. Another question is the amount of carbohydrates: Will it make sense to limit carbohydrates, since their amount coming from high-starch fruits/vegetables will be so high that the body will likely not switch to the faster use of fatty acids in these conditions? Will the Paleo diet regulate the acid–base body status (with many fruits and vegetables and a high amount of protein)?

## 5. Conclusions

The Paleo diet resulted in improvements in body weight and biomarkers linked to obesity, cardiovascular disease, type 2 diabetes, and metabolic syndrome. Even though short-term intervention showed some favorable effects, it can be argued that a short-term dietary intervention is too short to ensure stable effects. These improvements were mostly observed when the participants were only coached to change their dietary eating plan not to reduce body weight (ad libitum energy intake). Longer-term interventions are required to determine the continued effect of the Paleo diet (1) if a normoenergetic model of PD is applied, (2) if the reduction in body weight is maintained, especially under sports conditions, and (3) if enhanced sports performance is desired. The long-term consequences of these changes remain to be studied. Future research should focus on determining recommendations for athletes that embrace the positive aspects of the Paleo diet and minimize any deleterious impacts in terms of athletic performance. A longer follow-up and larger sample size are recommended in future clinical trials on the subject, in addition to a greater standardization of the Paleolithic diet used. In view of the positive short-term effects of the PD on health, and the lack of experiments on professional athletes, longer-term interventions are required to better determine the effect of the Paleo diet on athletic health status, body composition, and performance.

## Figures and Tables

**Table 1:** Articles included in the meta-analyses according to the author, the year of publications, the type of study, the characteristics of the populations undergoing interventions, and evaluated outcomes.

Study and Year	Type of Diets/ Type of Study	Subjects (n)/Participants Characteristic/Age/ Duration of Intervention (Time)	Effect on Body Composition	Effect on Lipid Profile	Effect on Blood Pressure	Effect on Carbohydrates Metabolism	Effect on Athletic Performance
Andersson et al. 2016 [45]	PD vs. CD (NNR) ad libitum RCT, parallel	[70] healthy postmenopausal women with body mass index (BMI) ≥ 27 kg/m^2^ (overweigh/obese) long-term (24 months)	Partially positive: Both diet groups decreased their BM and BMI at 6 and 24 months without significant differences between groups.	No effect: No difference in cholesterol levels over time or between groups.	Partially positive: BP was reduced at 6 months but returned to baseline levels at 24 months.	No effect: No differences were observed over time or between groups regarding fasting glucose, insulin concentrations and HOMA-IR.	Not analyzed
Boers et al. 2014 [53]	PD vs. CD (Dutch Health Cuncil) isocaloric	[32] men (*n* = 9) and women (*n* = 25) with at least two characteristics of the metabolic syndrome PD: 52 ± 10.2 CD: 55 ± 9 short-term (2 week)	Partially positive: In both groups, change was observed in WC.	Positive: Lower TC and TG and a higher mean HDL. The TC/HDL and TG/HDL ratios were lower in the PD compared to reference.	Positive: Lower SBP, DBP.	Positive: decreased fasting plasma insulin and HOMA-IR.	Not analyzed
Blomquist et al. 2017 [47]	PD vs. CD ad libitum RCT, parallel	[69] postmenopausal women with over- weight PD: 60 ± 5.6 CD: 61 ± 7 short-term (6 months); long-term (24 months)	Partially positive: Android fat decreased significantly more in the PD group during the first 6 months with weight maintenance at 24 months in both groups.	Positive: HDL levels increased in both groups between 6 and 24 months, LDL and TG levels decreased significantly in the PD group after 24 months.	Partially positive: In both groups, blood pressure decreased at 6 months, and the effect on DBP remained after 24 months in the PD group.	Partially positive: HOMA-IR decreased significantly in 6 months for PD group, rest of effect statistically insignificant.	Not analyzed
Blomquist et al. 2018 [48]	PD vs. CD ad libitum RCT, parallel	[58] postmenopausal women with over-weight/healthy PD: 60 ± 5.5 CD: 62 ± 5.7 short-term (6 months)	Positive: significantly larger reductions in body weight and SAG in PD compared to the CD group.	Partially positive: TG decreased significantly more in the PD group compared to the CD group. TC levels and LDL decreased in both groups, without differences between groups. The levels of HDL and FFA remained stable in both groups.	Not analyzed	Positive: The PD led to improved insulin sensitivity. The HOMA-IR index decreased significantly in the PD group, without significant difference between diet groups.	Not analyzed
Boraxbekk et al. 2015 [40]	PD vs. CD (NNR) ad libitum RCT, parallel	[20] overweight or obese postmenopausal women PD = 61 ± 1.6 CD = 61.6 ± 1.7 short-term (6 months)	Partially positive: PD and NNR improved anthropometric measurements without significant differences between groups.	Partially positive: Levels of FFA in plasma decreased in both groups.	Not analyzed	No effects: no changes in plasma glucose, insulin, or HOMA-IR.	Not analyzed
Fontes- Villalba et al. 2016 [51]	PD vs. CD (diabetes diet) ad libitum RCT, crossover	[13] patients with type 2 diabetes/ PD: 66 ± 6 CD: 63 ± 6 short-term (12 weeks)	Positive: weight loss was significantly greater after the PD than the diabetes diet	Not analyzed	Not analyzed	No effects: did not change fasting levels of insulin.	Not analyzed
Frassetto et al. 2009 [18]	3 days, three ramp-up diets of increasing potassium and fiber for 7 days, then a PD for 10 days; isocaloric RCT	[9] nonobese sedentary healthy volunteers/healthy population in physical activity 38 ± 12 short-term (10 day)	No effects: Not changed	Positive: large significant reductions in TC, LDL, and TG. No significant change occurred in HDL.	Positive: significant reductions in BP associated with improved arterial distensibility.	Positive: significant reduction in plasma insulin vs. time AUC during the OGTT.	Not analyzed
Genoni et al. 2016 [54]	PD vs. CD (AGHE) ad libitum RCT, parallel	[39] healthy women with BMI 27 ± 4 kg/m^2^ 47 ± 13 short-term (4 weeks)	Positive: Significantly greater BM loss and WC occurred in the PD.	Partially positive: In both dietary groups experienced within group reductions to TC and LDL. No significant differences in these changes between the dietary groups.	No effect: No significant differences.	No effect: No significant differences between dietary groups in biomarkers of metabolism (fasting glucose and insulin).	Not analyzed
Lindeberg et al. 2007 [28]	PD vs. CD (Mediterranean) ad libitum RCT, parallel	[29] ischaemic heart disease plus either glucose intolerance or type 2 diabetes PD: 65 ± 10 CD: 57 ± 7 short-term (12 weeks)	Positive: weight loss and a decrease in WC.	Not analyzed	Not analyzed	Positive: improving glucose tolerance.	Not analyzed
Masharani et al. 2015 [52]	PD vs. CD (ADA) isocaloric RCT, parallel	[24] type 2 diabetes patients PD: 58 ± 8 CD: 56 ± 13 short-term (2 weeks)	Partially positive: The average BM changes were similar in both groups without caloric restriction.	Positive: The PD group had statistically significant declines in TC, HDL, and LDL.	No effects: The mean BP did not significantly change in any of the two groups.	Positive: PD group had greater benefits on glucose control, with significant improvement in insulin sensitivity.	Not analyzed
Mellberg et al. 2014 [49]	PD vs. CD (NNR) ad libitum RCT, parallel	[69], (after 6 m. 61, after 2 y: 49) overweight postmenopausal women PD: 59.9 ± 5.5 CD: 60.3 ± 5.9 short-term (6 months); long-term (24 months)	Partially positive: Both groups significantly decreased FM at 6 months and 24 months, with a more pronounced loss in the PD at 6 months but not at 24 months. WC and SAG also decreased in both the groups, with a more pronounced decrease in the PD at 6 months.	Partially positive: TG levels decreased significantly more at 6 and 24 months in the PD than in the NNR. LDL and TC decreased at both 6 and 24 months, HDL increased in 24 months.	Positive: decreased in both 6 and 24 mons: DBP, SBP, HR.	No effect: No differences were measured over time or between groups with regard to fasting glucose and fasting insulin concentrations and tissue plasminogen activator activity.	Not analyzed
Otten et. al. 2016 [41]	PD vs. CD (LFD) ad libitum RCT	[41] healthy, overweight/obese postmenopausal women PD: 61 ± 6 CD: 66 ± 2 short-term (6 months); long-term (24 months)	Partially positive: Both diet groups decreased their BM, BMI, WC, and FM. At 6 months, the PD group showed a greater effect than the LFD group. The LFD lost less LBM compared with the PD. At 24 months, there were no significant differences in body composition between diet groups except for the better preservation of LBM in the LFD group.	Partially positive: TGs, TC, and LDL improved significantly more in the PD group during the first 6 months of the study. At 24 months, both study groups showed an improvement of HDL.	Partially positive: SBP improved in both study groups at 6 months. DBP improved only in the PD group.	Partially positive: HOMA-IR improved significantly after 6 months. Between 6 and 24 months, hepatic insulin sensitivity deteriorated significantly in the PD group with a similar trend in the LFD group.	Not analyzed
Österdahl et al. 2008 [56]	PD vs. CD (normal diet) ad libitum Short communication	[14] healthy volunteers 30 ± 10 short-term (3 weeks)	Positive: Mean BM and WC decreased.	No effects: Not changed	Partially positive: Decreased SBP	No effects: Not changed	Not analyzed
Pastore et. al. 2015 [39]	PD vs. CD (AHA) ad libitum 4 months CD, followed by 4 months PD; RCT	[20] hypercholesterolemic patients 53 ± 7 short-term (4 months)	Positive: PD induced a significant BM loss, compared with AHA.	Positive: PD significantly lowered mean TC, LDL, and TG and increased HDL.	Not analyzed	Not analyzed	Not analyzed
Stomby et al. 2015 [46]	PD vs. CD (NNR) ad libitum RCT, parallel	[49] overweight and obese postmenopausal women short-term (6 months) long-term (24 months)	Partially positive: At 6 months, the PD group had a greater reduction of BM, BMI, and FM. At 24 months, there were no significant differences in anthropometric measurements between the groups.	Partially positive: TC decreased after 6 months but was unaltered at 24 months compared with baseline. TGs and LDL decreased throughout the intervention, whereas HDL increased after 24 months. There were no group differences in blood lipids.	Partially positive: SBP and DBP decreased at 6 months but had increased to baseline levels after 24 months. There were no group differences in blood pressure.	Partially positive: Fasting serum insulin and HOMA-IR decreased at 6 months but was unaltered after 24 months. There were no group differences, fasting serum insulin, and HOMA-IR.	Not analyzed
Otten et al. 2017 [42]	PD vs. PD-EX (PD-EX: a combination of aerobic exercise and resistance training in 1 h sessions three times weekly) ad libitum	[29] individuals with type 2 diabetes with BMI 25–40 kg/m^2^ short-term (12 weeks)	Positive: Both groups showed decreases in BM, FM and WC, without differences between intervention groups. Male participants decreased their WC more in the PD group compared to the PD-EX. Males in the PD-EX group retained more LBM than males in the PD group.	Partially positive: TG decreased in both study groups, while the HDL and LDL levels remained unchanged throughout the intervention.	Positive: Blood pressure decreased during the study in both intervention groups without any group difference.	Positive: Insulin sensitivity and glycemic control improved in both groups, without a difference between groups. The HOMA-IR and revised QUICKI improved in both intervention groups, and the HbA1c decreased during the study in both the PD group and the PD-EX group.	Partially positive The VO_2_max and the ergometer cycling workload increased during the study in the PD-EX group, but not in the PD group. Resting HR decreased more in the PD-EX group than the PD group.
Otten et al. 2018 [43]	PD vs. PD-EX (PD-EX: aerobic exercise/resistance training in 1 h sessions 3 times weekly) ad libitum RCT	[32] individuals with type 2 diabetes with BMI 25–40 kg/m^2^ short-term (12 weeks)	Positive: Both study groups showed a BM, BMI, FM loss.	Positive: TG decreased in both gropus	Not analyzed	Positive: Both groups improved their peripheral and adipose tissue insulin sensitivity, but not their hepatic insulin sensitivity.	Partially positive: The VO_2_max increased in the PD-EX group only.
Otten et al. 2019 [44]	PD vs. PD-EX (exercise training 3 h per week) ad libitum	[22] overweight and obese subjects with type 2 diabetes mellitus PD: 59 PD-EX: 61 short-term (12 weeks)	Positive: significant decreases in both groups in terms of BM, BMI, WC	Partially positive: The PD-EX group showed significant decreases in myocardial TG levels. These variables were unchanged in the PD group. There were significant decreases in both groups in terms of fasting triglycerides.	Positive: significant decreases in both groups in terms of SBP and DBP.	Positive: significant decreases in both groups in terms of fasting glucose, HbA1c, fasting insulin, and HOMA-IR.	Partially positive The VO_2_max increased significantly in both groups, although the increase in the PD-EX group was more pronounced. Mean resting HR decreased and the W max. increased significantly in the PD-EX group, while no changes were seen for these measures in the PD group.
Markofski et al. 2019 [71]	PD pre vs. post ad libitum	[7] overweight, physically inactive but otherwise healthy adults 32.7 ± 4.9 short-term (8 weeks)	Positive: time effect pre- to post-intervention for BM and BMI.	Not analyzed	No effect: SBP and DPB were unchanged following the PD intervention.	Not described	Not analyzed
Ryberg et al. 2013 [37]	PD pre vs. post ad libitum higher energy intake before intervention	[10] healthy, nonsmoking postmenopausal women with BMI > 27 kg/m^2^ short-term (5 weeks)	Positive: BMI, waist and hip circumference, waist/hip ratio, and SAG also decreased significantly.	Positive: TC, TG, HDL, LDL, and LDL/HDL decreased significantly.	Positive: DBP and resting HR decreased significantly.	Partially positive: Fasting serum glucose and HOMA indices decreased significantly. Insulin sensitivity did not change.	Not analyzed
Smith et al. 2014 [55]	PD pre vs. post subjects completed a CrossFit-based exercise program while adhering to the Paleo diet. ad libitum	[44] healthy population F: 31.2 M: 33.5 short-term (10 weeks)	Positive: FM percentage decreased significantly, as did BM.	Partially positive: A significant increase in non-HDL, LDL, TC/HDL, and TC in healthy subjects following a PD. Deleterious changes were found in those with optimal HDL, non-HDL, TC/HDL, and LDL whereas those within sub-optimal stratifications showed no significant change.	Not analyzed	Not analyzed	Positive: significantly increasing the VO_2_max, a common measure of cardiorespiratory fitness

Android fat—fat accumulates around the central trunk region; ADA—American Diabetes Association; AGHE—Australian Guide to Healthy Eating; AHA—American Heart Association; AUC—area under the curve; BM—body mass; BMI—body mass index; BP—blood pressure; CD—control diets; DBP—diastolic blood pressure; FFA—free fatty acid; FM—fat mass; fP—fasting plasma; HbA1c—glycated hemoglobin; HDL—high-density lipoprotein cholesterol; HOMA-IR—homeostasis model assessment of insulin resistance; HR—heart rate; LBM—lean body mass; LDL—low-density lipoprotein cholesterol; LFD—low-fat diet; NNR—Nordic Nutrition Recommendation; OGTT—oral glucose tolerance test; PD—Paleo diet; PD-EX—Paleo diet with exercise training; QUICKI—quantitative insulin sensitivity check index; RCT—randomized control trial; SAG—sagittal abdominal diameter; SBP—systolic blood pressure; TC—total cholesterol; TG—triglycerides; VO_2_max—maximum oxygen uptake; W max—maximum workload; WC—waist circumference.

**Table 2:** Changes in body composition (body mass, body mass index, waist circumference, and fat mass in kg and %) in short-term PDs, PD, and CD studies (up to 6 months).

	BM (kg)			BMI (kg/m^2^)			WC (cm)			FM (kg)			FM (%)		
Indicator	PDs	PD	CD	PDs	PD	CD	PDs	PD	CD	PDs	PD	CD	PDs	PD	CD
beta	−5.798	−5.321	−3.916	−2.084	−3.076	−1.697	−5.007	−4.271	−3.114	−4.510	−4.125	−2.136	−2.391	−2.557	−0.281
95%HCI	−4.269	−3.163	−2.579	−1.361	−2.560	−0.989	−3.132	−2.977	−2.429	−1.572	0.391	1.234	−1.158	0.128	1.292
95%LCI	−7.328	−7.478	−5.254	−2.807	−3.592	−2.406	−6.882	−5.564	−3.798	−7.448	−8.642	−5.506	−3.625	−5.242	−1.866
*p* value	0.000	0.000	0.000	0.000	0.000	0.000	0.000	0.000	0.000	0.003	0.073	0.214	0.000	0.062	0.722
K	16	8	8	8	3	3	9	5	5	3	2	2	4	2	2

Beta: the size of the average effect (within group); 95% HCI and 95% LCI: limits of 95% confidence interval of the effect; *p*-value: significance level of the intra-group effect. Results statistically significant at least at the 0.1 significance level are marked red; k: number of studies included; PDs: experimental group (both PD vs. CD and PD pre vs. post; PD vs. PD + EX); PD: experimental group limited only to PD vs. CD studies; CD: control group.

**Table 3:** Changes in body composition (body mass, body mass index, waist circumference, and fat mass in kg and %) in long-term PDs, PD, and CD studies (over 6 months).

	BM (kg)			BMI (kg/m^2^)			WC (cm)			FM (kg)			FM (%)		
Indicator	PDs	PD	CD	PDs	PD	CD	PDs	PD	CD	PDs	PD	CD	PDs	PD	CD
Beta	−8.690	−8.690	−5.760	−2.765	−2.765	−1.815	−12.097	−12.097	−10.874	−5.464	−5.464	−4.451	−2.722	−2.722	−2.607
95%HCI	−6.070	−6.070	−4.283	−1.937	−1.937	−1.435	−7.624	−7.624	−7.640	−3.441	−3.441	−2.889	−1.323	−1.323	−0.762
95%LCI	−11.310	−11.310	−7.236	−3.594	−3.594	−2.196	−16.570	−16.570	−14.108	−7.488	−7.488	−6.013	−4.120	−4.120	−4.452
*p* value	0.000	0.000	0.000	0.000	0.000	0.000	0.000	0.000	0.000	0.000	0.000	0.000	0.000	0.000	0.006
k	3	3	3	3	3	3	2	2	2	2	2	2	2	2	2

Beta: the size of the average effect (within group); 95% HCI and 95% LCI: limits of 95% confidence interval of the effect; *p*-value: significance level of the intra-group effect. Results statistically significant at least at the 0.1 significance level are marked red; k: number of studies included; PDs: experimental group (both PD vs. CD and PD pre vs. post; PD vs. PD + EX); PD: experimental group limited only to PD vs. CD studies; CD: control group.

**Table 4:** Changes in lipid profile (total cholesterol, triglycerides, HDL-C, and LDL-C) in short-term PDs, PD, and CD studies (up to 6 months).

	TC (mg/dL)			TG (mg/dL)			HDL-C (mg/dL)			LDL-C (mg/dL)		
Indicator	PDs	PD	CD	PDs	PD	CD	PDs	PD	CD	PDs	PDs	CD
Beta	−0.576	−0.657	−0.336	−0.341	−0.302	−0.089	−0.002	−0.078	−0.072	−0.373	−0.408	−0.227
95%HCI	−0.387	−0.544	−0.246	−0.222	−0.156	−0.044	0.104	−0.017	−0.003	−0.189	−0.318	−0.147
95%LCI	−0.766	−0.770	−0.427	−0.460	−0.448	−0.134	−0.108	−0.139	−0.142	−0.557	−0.498	−0.308
*p* value	0.000	0.000	0.000	0.000	0.000	0.000	0.971	0.012	0.042	0.000	0.000	0.000
k	14	8	8	14	8	8	13	7	7	13	7	7

Beta: the size of the average effect (within group); 95% HCI and 95% LCI: limits of 95% confidence interval of the effect; *p*-value: significance level of the intra-group effect. Results statistically significant at least at the 0.1 significance level are marked red; k: number of studies included; PDs: experimental group (both PD vs. CD and PD pre vs. post; PD vs. PD + EX); PD: experimental group limited only to PD vs. CD studies; CD: control group.

**Table 5:** Changes in lipid profile (total cholesterol, triglycerides, HDL-C, and LDL-C in long-term PDs, PD, and CD studies (over 6 months).

	TC (mg/dL)			TG (mg/dL)			HDL-C (mg/dL)			LDL-C (mg/dL)		
Indicator	PDs	PD	CD	PDs	PD	CD	PDs	PD	CD	PDs	PD	CD
Beta	−0.228	−0.228	0.010	−0.225	−0.225	−0.077	0.181	0.181	0.205	−0.307	−0.307	−0.085
95%HCI	−0.076	−0.076	0.051	−0.148	−0.148	−0.004	0.235	0.235	0.263	−0.203	−0.203	0.037
95%LCI	−0.381	−0.381	−0.031	−0.302	−0.302	−0.151	0.128	0.128	0.147	−0.412	−0.412	−0.208
*p* value	0.003	0.003	0.639	0.000	0.000	0.040	0.000	0.000	0.000	0.000	0.000	0.172
k	4	4	4	4	4	4	3	3	3	3	3	3

Beta: the size of the average effect (within group); 95% HCI and 95% LCI−limits of 95% confidence interval of the effect; *p*-value: significance level of the intra-group effect. Results statistically significant at least at the 0.1 significance level are marked red; k: number of studies included; PDs: experimental group (both PD vs. CD and PD pre vs. post; PD vs. PD + EX); PD: experimental group limited only to PD vs. CD studies; CD: control group.

**Table 6:** Changes in blood pressure (systolic blood pressure, diastolic blood pressure, and heart rate) in short-term PDs, PD, and CD studies (up to 6 months).

	SBP (mmHg)			DBP (mmHg)			HR (bpm)		
Indicator	PDs	PD	CD	PDs	PD	CD	PDs	PD	CD
Beta	−6.926	−8.493	−5.579	−4.922	−5.345	−0.848	−3.021	−2.200	−3.200
95%HCI	−4.484	−5.467	−2.654	−3.247	−3.047	2.513	0.906	−1.729	−2.785
95%LCI	−9.367	−11.519	−8.504	−6.597	−7.643	−4.210	−6.948	−2.671	−3.615
*p* value	0.000	0.000	0.000	0.000	0.000	0.621	0.132	0.000	0.000
k	12	6	6	12	6	6	4	1	1

Beta: the size of the average effect (within group); 95% HCI and 95% LCI−limits of 95% confidence interval of the effect; *p*-value: significance level of the intra-group effect. Results statistically significant at least at the 0.1 significance level are marked red; k: number of studies included; PDs: experimental group (both PD vs. CD and PD pre vs. post; PD vs. PD + EX); PD: experimental group limited only to PD vs. CD studies; CD: control group.

**Table 7:** Changes in blood pressure (systolic blood pressure and diastolic blood pressure) in long-term PDs, PD, and CD studies (over 6 months).

	SBP (mmHg)			DBP (mmHg)		
Indicator	PDs	PD	CD	PDs	PD	CD
Beta	−1.021	−1.021	0.828	−4.613	−4.613	0.040
95%HCI	1.785	1.785	2.479	−3.837	−3.837	4.315
95%LCI	−3.827	−3.827	−0.822	−5.389	−5.389	−4.234
*p* value	0.476	0.476	0.325	0.000	0.000	0.985
k	3	3	3	3	3	3

Beta: the size of the average effect (within group); 95% HCI and 95% LCI: limits of 95% confidence interval of the effect; *p*-value: significance level of the intra-group effect. Results statistically significant at least at the 0.1 significance level are marked red; k: number of studies included; PDs: experimental group (both PD vs. CD and PD pre vs. post; PD vs. PD + EX); PD: experimental group limited only to PD vs. CD studies; CD: control group.

**Table 8:** Changes in carbohydrate metabolism (fP glucose, fP insulin, HOMA-IR, and HbA1c) in short-term PDs, PD, and CD studies (up to 6 months).

	fP Glucose (mmol/L)			fP Insulin (mmol/L)			HOMA-IR			HbA1c (%)		
Indicator	PDs	PD	CD	PDs	PD	CD	PDs	PD	CD	PDs	PD	CD
beta	−0.511	−0.450	−0.167	−1.922	−1.600	−0.230	−0.442	−0.408	−0.074	−0.404	−0.178	−0.122
95%HCI	−0.176	−0.025	0.068	−0.916	−0.323	0.224	−0.196	−0.185	0.037	−0.019	−0.028	0.021
95%LCI	−0.847	−0.876	−0.40	−2.928	−2.878	−0.684	−0.687	−0.631	−0.185	−0.831	−0.328	−0.265
*p* value	0.003	0.050	0.165	0.000	0.011	0.267	0.000	0.000	0.157	0.063	0.020	0.095
k	12	8	7	11	5	5	9	5	5	3	2	2

Beta: the size of the average effect (within group); 95% HCI and 95% LCI: limits of 95% confidence interval of the effect; *p*-value: significance level of the intra-group effect. Results statistically significant at least at the 0.1 significance level are marked red; k: number of studies included; PDs: experimental group (both PD vs. CD and PD pre vs. post; PD vs. PD + EX); PD: experimental group limited only to PD vs. CD studies; CD: control group.

**Table 9:** Changes in carbohydrate metabolism (fP glucose, fP insulin, and HOMA-IR) in long-term PDs, PD, and CD studies (over 6 months).

	fP Glucose (mmol/L)			fP Insulin (mmol/L)			HOMA-IR		
Indicator	PDs	PD	CD	PDs	PD	CD	PDs	PD	CD
beta	−0.113	−0.113	0.002	−0.240	−0.240	0.351	−0.022	−0.022	0.304
95%HCI	0.169	0.169	0.115	0.304	0.304	1.186	0.109	0.109	0.523
95%LCI	−0.396	−0.396	−0.111	−0.784	−0.784	−0.484	−0.153	−0.153	0.085
*p* value	0.431	0.431	0.973	0.387	0.387	0.410	0.741	0.741	0.007
k	2	2	2	2	2	2	3	3	3

Beta: the size of the average effect (within group); 95% HCI and 95% LCI: limits of 95% confidence interval of the effect; *p*-value: significance level of the intra-group effect Results statistically significant at least at the 0.1 significance level are marked red; k: number of studies included; PDs: experimental group (both PD vs. CD and PD pre vs. post; PD vs. PD + EX); PD: experimental group limited only to PD vs. CD studies; CD: control group.

**Table 10:** Changes in physical capacity (VO_2_max; Maximum Workload) in PD, PDs, and PD-EX.

	VO_2_max (mL/kg/min)			VO_2_max (L/min)			Maximum Workload (W max)		
Indicator	PDs	PD	PD-EX	PDs	PD	PD-EX	PDs	PD	PD-EX
Beta	2.068	1.921	3.484	0.127	0.000	0.400	0.000	0.000	30.000
95%HCI	2.853	2.725	4.940	0.400	0.112	0.754	0.112	0.112	56.575
95%LCI	1.282	1.116	2.028	−0.146	−0.112	0.046	−0.112	−0.112	3.425
*p* value	0.000	0.000	0.000	0.362	1.000	0.027	1.000	1.000	0.027
k	3	2	2	2	1	1	1	1	1

Beta: the size of the average effect (within group); 95% HCI and 95% LCI: limits of 95% confidence interval of the effect; *p*-value: significance level of the intra-group effect. Results statistically significant at least at the 0.1 significance level are marked red; k: number of studies included; PD: experimental group (PD group results both for PD vs. PD-EX and PD pre vs. post studies); PDs: PD group limited only to PD vs. PD-EX studies; PD-EX—paleo diet with supervised training.

## Data Availability

Not applicable.
